# Third‐wave cognitive behaviour therapies for weight management: A systematic review and network meta‐analysis

**DOI:** 10.1111/obr.13013

**Published:** 2020-03-17

**Authors:** Emma R. Lawlor, Nazrul Islam, Sarah Bates, Simon J. Griffin, Andrew J. Hill, Carly A. Hughes, Stephen J. Sharp, Amy L. Ahern

**Affiliations:** ^1^ MRC Epidemiology Unit University of Cambridge Cambridge UK; ^2^ School of Health and Related Research, Faculty of Medicine, Dentistry and Health University of Sheffield Sheffield UK; ^3^ Primary Care Unit, Institute of Public Health University of Cambridge Cambridge UK; ^4^ Division of Psychological and Social Medicine, School of Medicine University of Leeds Leeds UK; ^5^ Fakenham Medical Practice Norfolk UK; ^6^ Norwich Medical School University of East Anglia Norwich UK

**Keywords:** network meta‐analysis, obesity, third‐wave behavioural therapy, weight loss

## Abstract

This systematic review and network meta‐analysis synthesized evidence on the effects of third‐wave cognitive behaviour therapies (3wCBT) on body weight, and psychological and physical health outcomes in adults with overweight or obesity. Studies that included a 3wCBT for the purposes of weight management and measured weight or body mass index (BMI) pre‐intervention and ≥ 3 months post‐baseline were identified through database searches (MEDLINE, CINAHL, Embase, Cochrane database [CENTRAL], PsycINFO, AMED, ASSIA, and Web of Science). Thirty‐seven studies were eligible; 21 were randomized controlled trials (RCT) and included in the network meta‐analyses. Risk of bias was assessed using RoB2, and evidence quality was assessed using GRADE. Random‐effects pairwise meta‐analysis found moderate‐ to high‐quality evidence suggesting that 3wCBT had greater weight loss than standard behavioural treatment (SBT) at post‐intervention (standardized mean difference [SMD]: −0.09, 95% confidence interval [CI]: −0.22, 0.04; N = 19; I^2^ = 32%), 12 months (SMD: −0.17, 95% CI: −0.36, 0.02; N = 5; I^2^ = 33%), and 24 months (SMD: −0.21, 95% CI: −0.42, 0.00; N = 2; I^2^ = 0%). Network meta‐analysis compared the relative effectiveness of different types of 3wCBT that were not tested in head‐to‐head trials up to 18 months. Acceptance and commitment therapy (ACT)‐based interventions had the most consistent evidence of effectiveness. Only ACT had RCT evidence of effectiveness beyond 18 months. Meta‐regression did not identify any specific intervention characteristics (dose, duration, delivery) that were associated with greater weight loss. Evidence supports the use of 3wCBT for weight management, specifically ACT. Larger trials with long‐term follow‐up are needed to identify who these interventions work for, their most effective components, and the most cost‐effective method of delivery.

**Abbreviations**
3wCBT: third‐wave cognitive behaviour therapy
ACT: acceptance and commitment therapy
BMI: body mass index
CBT: cognitive behaviour therapy
CFT: compassion focused therapy
CI: confidence intervals
CONSORT: Consolidated Standards of Reporting Trials
DBT: dialectical behaviour therapy
GRADE: Grading of Recommendations, Assessment, Development and Evaluations
MBCT: mindfulness‐based cognitive behavioural therapy
RCT: randomized controlled trial
ROBINS‐I: Risk Of Bias In Non‐randomised Studies of Interventions
RoB2: Risk of Bias 2
SBT: standard behavioural treatment
SD: standard deviation
SMD: standardized mean difference
TIDieR: Template for Intervention Description and Replication

## 1 BACKGROUND

Although behavioural interventions are effective at helping people to lose weight, many people struggle to sustain effective weight management behaviours over extended periods due to a combination of biological, psychological, social, and environmental factors that drive weight gain.^1^, ^2^ Standard behavioural programmes can be effective in the short term, but less so in the longer term.^3^, ^4^, ^5^, ^6^ These usually combine diet and physical activity advice with core behavioural change techniques including goal setting, self‐monitoring, problem solving, and planned social support.^7^ It has been proposed that third‐wave cognitive behaviour therapies (3wCBT), including acceptance and commitment therapy (ACT), dialectical behaviour therapy (DBT), mindfulness‐based cognitive behavioural therapy (MBCT), and compassion‐focused therapy (CFT),^8^, ^9^, ^10^ may have better short‐ and long‐term outcomes.^2^

The theoretical case for 3wCBT for weight management has been well articulated.^2^ In brief, these therapies encourage people to accept aversive internal experiences (eg, food cravings, physical discomfort) rather than avoid them. Increased present‐moment, non‐judgemental awareness and psychological flexibility may assist an individual in recognizing internal and external cues to overeat and alter behavioural responses to be more in line with their values. Fostering a compassionate attitude towards the self could also help prevent discouragement following minor lapses.^2^, ^8^, ^9^

However, the evidence of their superior effectiveness is less clear. Previous systematic reviews and meta‐analyses primarily focused on mindfulness‐ and/or acceptance‐based interventions.^11^, ^12^, ^13^, ^14^, ^15^, ^16^, ^17^, ^18^ Three reviews^13^, ^15^, ^16^ have reported a quantitative synthesis of pre‐intervention to post‐intervention change without comparing the effect against a comparator. Two of these three reviews reported a “small” pre‐intervention to post‐intervention change in weight^15^ or body mass index (BMI)^13^ while the other study^16^ reported a null effect on BMI. Critically, only one review^14^ reported a meta‐analytic synthesis that compared the effectiveness of mindfulness‐ and acceptance‐based interventions with those in other active interventions and control arms using appropriate statistical methods. A small but significant difference in weight or BMI was reported at post‐intervention, favouring mindfulness and acceptance‐based interventions over comparator arms. Subgroup analysis suggested that the effect may only hold when the comparator is waitlist control. In that review, there was no restriction on the minimum follow‐up duration and outcomes were analysed at 1‐month post‐intervention (or the closest measurement to this). Thus, the pooled estimates reflected a mix of very short‐term and longer term effects. Moreover, without a restriction on minimum BMI, these findings are less relevant from a policy perspective because behavioural weight management programmes are intended for adults with overweight/obesity.^19^, ^20^ This concern is compounded by the finding that a lower BMI was associated with a larger effect size.

To our knowledge, no head‐to‐head trial exists that has compared the effectiveness of different types of 3wCBT on weight management. In the absence of head‐to‐head trials, network meta‐analysis can estimate the indirect evidence on the comparative effectiveness of different types of 3wCBT. The proposed mechanism for the superior effects of 3wCBT is through improvements in eating behaviour and psychological outcomes, so it is also important to synthesize evidence on the impact of 3wCBT on these outcomes. In addition, evidence synthesis of the effect of 3wCBT on eating behaviour and psychological outcomes has been limited to pre‐intervention to post‐intervention change^13^, ^15^, ^16^ and has not considered longer follow‐up periods.

To address these knowledge gaps, we conducted the most comprehensive, inclusive, and relevant review and quantitative synthesis of available evidence to date. We included different types of 3wCBT beyond mindfulness and acceptance‐based interventions. Our main objectives were (a) to evaluate the effectiveness of 3wCBTs on weight management by pooling the pre‐intervention to post‐intervention change effect estimates across all study types, (b) to compare the effectiveness of 3wCBTs on weight management against no/minimal interventions and standard behavioural treatment (SBT) separately using random‐effects pairwise meta‐analysis of randomized control trials (RCTs), (c) to estimate the comparative effectiveness of different types of 3wCBTs on weight management using random‐effects network meta‐analysis of RCTs, (d) to evaluate the impact of 3wCBT on eating behaviour and psychological and physical health outcomes, and (e) to provide a detailed description of intervention characteristics and to identify whether any of these are associated with better weight change outcomes by using meta‐regression.

## 2 METHODS

### 2.1 Protocol and registration

The protocol was registered on Prospero (CRD42018088255) prior to article screening.^21^

### 2.2 Eligibility criteria

Participants were community‐dwelling adults (≥18 years) with overweight or obesity (BMI ≥25 kg/m^2^) seeking assistance with weight management. Studies had to include a 3wCBT intervention for the purpose of weight management. Multi‐component interventions (eg, including diet and physical activity advice) were acceptable, with no restriction placed on the proportion of the intervention using 3wCBT. Interventions could be of any duration. Comparisons were (a) no/minimal intervention, (b) SBT, or (c) no comparator (single‐arm pre‐intervention to post‐intervention studies). We defined SBT as structured programmes providing diet and/or physical activity advice and standard behaviour change techniques (eg, goal setting, self‐monitoring, problem solving, social support). The primary outcome was body weight or BMI. Studies needed to measure this pre‐intervention and at least 3‐months post‐baseline. Secondary outcomes were stress, anxiety, depression, meta‐cognition, eating attitudes, eating behaviours, body satisfaction, quality of life, blood pressure, lipids, glycaemia, and adherence to treatment. All outcomes reported at 3‐months from baseline and beyond were extracted. All settings apart from laboratories were eligible. We included research articles, theses, and dissertations reporting RCTs, non‐RCTs, prospective cohort and case series studies.

### 2.3 Information sources

Databases (MEDLINE, CINAHL, Embase, Cochrane database [CENTRAL], PsycINFO, AMED, ASSIA, and Web of Science) were searched by ERL from inception with no restrictions, using keywords and subject heading searches relating to the concepts: (a) third‐wave CBTs and (b) overweight, obesity, or weight management (see Table S1). The initial search was conducted on 16 January 2018, and an updated search was conducted on 25 September 2019. Reference lists of eligible studies and relevant reviews were searched, and authors of relevant abstracts were contacted to identify whether findings had been accepted for publication.

### 2.4 Study selection

Titles and abstracts, then full texts, were screened independently by two of three researchers, with a third reviewer adjudicating uncertainty or disagreement. Study authors were contacted to resolve any questions about eligibility. Non‐English language texts were translated into English by colleagues who were fluent in that language.

### 2.5 Data collection process

Data were extracted independently by two of four researchers using a form based on the Cochrane data extraction form,^22^ the Consolidated Standards of Reporting Trials (CONSORT) 2010 statement,^23^ and the Template for Intervention Description and Replication (TIDieR) checklist^24^ and cross‐checked for consistency. Attempts were made to contact authors to retrieve missing data. If there was no response after two attempts, we used the data available in the published work.

### 2.6 Risk of bias

Two researchers assessed studies independently using the Risk of Bias 2 tool (RoB 2)^25^ or the Risk Of Bias In Non‐randomized Studies of Interventions tool (ROBINS‐I),^26^ dependent upon study design. A third reviewer adjudicated inconsistency. The quality of evidence was assessed using the Grading of Recommendations Assessment, Development and Evaluation (GRADE) approach, which classifies studies as “high”, “moderate”, “low”, or “very low” quality.^27^

### 2.7 Missing data

For the primary outcomes, where standard deviations (SDs) for mean change were missing and not provided following author correspondence, these were imputed using the following methods, in order of prioritization: (a) imputed from other time points within same study, (b) estimated from *t* statistics, Cohen's *d*, p‐values or confidence intervals (CIs),^28^, ^29^, ^30^, ^31^, ^32^ (c) estimated using a correlation coefficient of .97, based on empirical data from seven studies^28^, ^30^, ^33^, ^34^, ^35^, ^36^, ^37^ (17 estimates) that reported SDs for baseline, follow‐up, and mean change.^38^, ^39^, ^40^, ^41^, ^42^, ^43^, ^44^, ^45^, ^46^ Insufficient data prevented this approach for secondary outcomes, so we used a correlation of .7 as in previous studies.^47^, ^48^

### 2.8 Synthesis of results

Stata/SE v.14.2^49^ was used for all statistical analyses. Following guidance,^50^ we focused on 95% CIs, rather than statistical significance. For example, unlike conventional interpretations, we did not outright interpret an effect estimate “non‐significant” if the lower or upper bound of the 95% CI was slightly above/below the null value; we interpreted them as “suggestive” of an effect.

#### 2.8.1 Pooled estimates of intervention‐specific effects from all study types

Intervention‐specific effects (post‐intervention minus pre‐intervention) were estimated by pooling effect estimates from all study designs. Due to heterogeneity in outcome measurement, effect estimates were reported as standardized mean change from the random‐effects meta‐analysis.^51^ Effect estimates were reported at the earliest measurement post‐intervention (≥3 months from baseline) and at 3, 6, 9, 12, 18, 24, and 36 months from baseline. Outcomes falling between these time points were included with the closest time point.

#### 2.8.2 Intervention comparisons: Direct evidence from pairwise meta‐analysis of RCTs

The direct effect comparing 3wCBT against (a) no/minimal intervention and (b) SBT was estimated using random‐effects^51^ pairwise meta‐analysis of RCTs. The standardized mean difference (SMD) calculated using Hedges' method and 95% CI were reported.^52^

#### 2.8.3 Intervention comparisons: Indirect and mixed evidence from network meta‐analysis of RCTs

To compare types of 3wCBT, random‐effects network meta‐analysis of RCTs was conducted to estimate the indirect and mixed (direct plus indirect) evidence.^53^ Basic assumptions were checked conceptually and statistically.^53^ For example, to avoid violating the transitivity assumption, which requires that the comparator arm (eg, the waitlist control) is comparable across the trials, the comparators (SBT and no/minimal intervention) were not pooled/used together. Similarly, the intervention arms were dropped (namely resistance exercise^35^ and food environment modification^54^) if they were not comparable with other intervention arms. The consistency assumption was checked statistically to see if the direct and indirect effect estimates were comparable enough to pool them together into the mixed evidence.^55^, ^56^ Effect estimates were reported as SMD and 95% CIs. The relative ranking probability of each intervention being the best treatment was estimated using rankograms.^57^

#### 2.8.4 Sensitivity analysis

The influence of individual studies on weight change of 3wCBT compared with no/minimal intervention or SBT was examined using influence plots, where one study was removed at a time to see its effect on the overall estimate.^58^

#### 2.8.5 Meta‐regression on intervention and study characteristics

Where at least 10 studies provided relevant data,^59^ meta‐regression was used to identify potential sources of heterogeneity for pre‐specified characteristics: number of sessions (continuous; <12 vs. ≥12 sessions), duration of intervention (<3 months vs. ≥3 months, <6 months vs. ≥6 months), method of delivery (face‐to‐face vs. remote; group vs. individual), and risk of bias (low, some concerns, high).

#### 2.8.6 Secondary outcomes

Only a small number of studies reported the secondary outcomes at each follow‐up time point, so the first time point post‐intervention was used and network meta‐analysis was not conducted. Pooled and pairwise meta‐analyses were conducted for secondary outcomes reported in two or more studies.

For consistency, we defined “change” as post‐intervention minus pre‐intervention values, so a negative change estimate indicates that the outcome decreased after the intervention.

## 3 RESULTS

**Figure 1:**
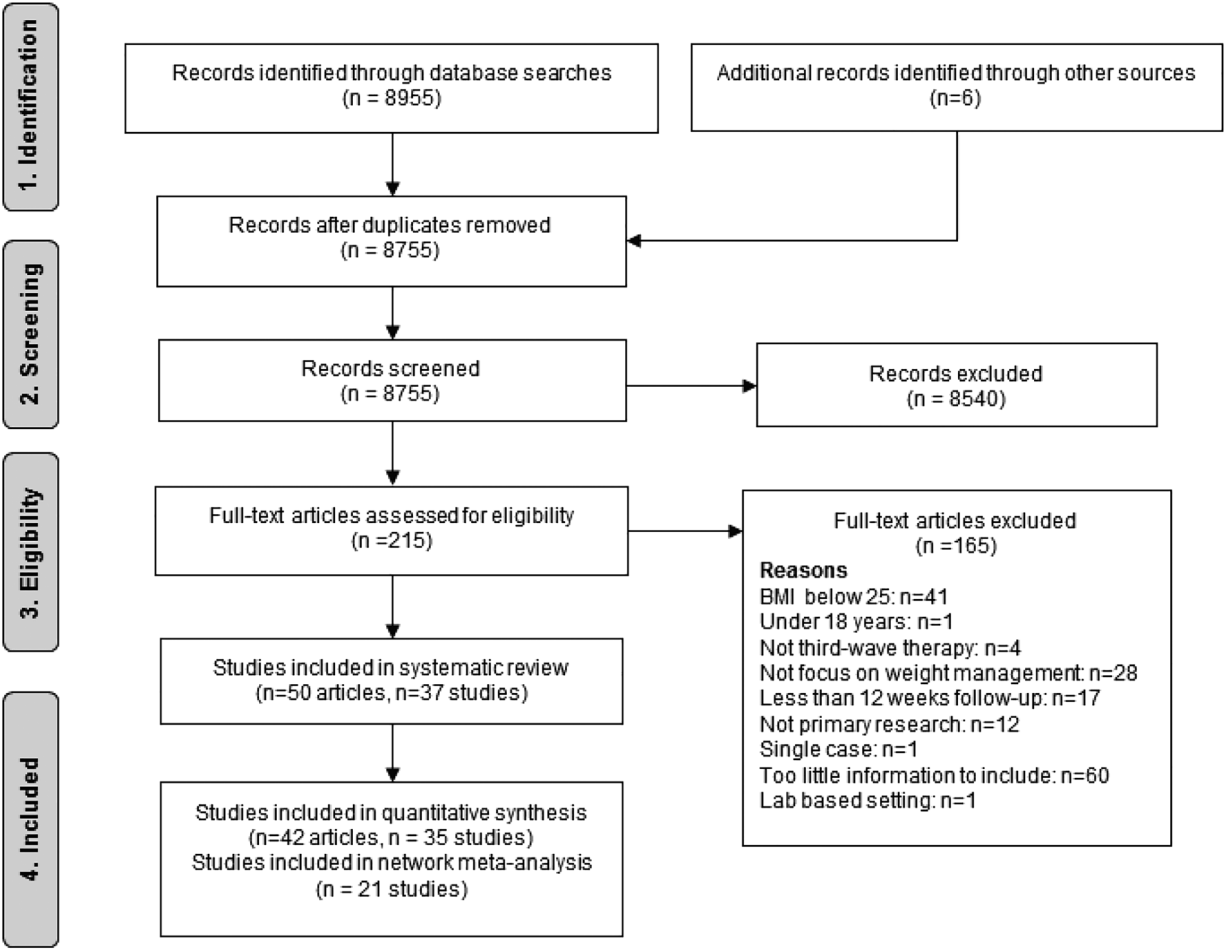
PRISMA flow diagram

After duplicate removal, 8755 titles and abstracts were screened and 215 full text articles were assessed. Two additional studies and four articles related to already included studies were identified from reference lists^60^, ^61^, ^62^, ^63^, ^64^ and contacting an author.^65^ Fifty articles reporting 37 studies met the inclusion criteria (Figure 1). Two studies were excluded from the meta‐analyses due to cointerventions (pharmacotherapy^66^ and bariatric surgery^39^). Thirty‐five studies were used in the pooled‐effects meta‐analysis of pre‐intervention to post‐intervention changes, and the 21 RCT design studies were used in the network meta‐analysis that compared different interventions.

### 3.1 Study characteristics

Seventeen studies^30^, ^31^, ^32^, ^33^, ^36^, ^41^, ^45^, ^46^, ^66^, ^67^, ^68^, ^69^, ^70^, ^71^, ^72^, ^73^, ^74^ used a two‐group RCT, four^35^, ^40^, ^44^, ^54^ used a three‐group RCT, and one used a two‐group cluster RCT design.^42^ Fourteen studies^28^, ^29^, ^34^, ^38^, ^43^, ^64^, ^65^, ^75^, ^76^, ^77^, ^78^, ^79^, ^80^ used a pre‐intervention to post‐intervention one‐group design, and one study was a non‐randomized three‐group study.^39^ The majority of studies were conducted in the United States (n = 28). The other studies were conducted in New Zealand,^34^ Italy,^39^ United Kingdom,^41^, ^42^, ^77^ the Netherlands,^43^, ^64^ Finland,^44^ and Portugal^70^ (Table 1).

**Table 1:** Characteristics of included studies

	Population				Intervention				Comparison	Outcomes	
First author, year	N	Age, years^a^	BMI, kg/m^2a^	Female; N, %	Intervention(s)	Primary delivery mode	Group or individual	Length, months	Comparison	Measurements	Time points, months^b^
*Randomized controlled trials*											
Blevins, 2008^33^	41	20.7 (1.4)	29.6 (1.9)	41 (100)	MBCT	Face to face	Group	2	SBT	Weight; anxiety; depression; binge eating; body dissatisfaction	2, 5
Carpenter, 2017^74^	75	47.3 (10.0)	31.5 (2.3)	69 (92)	MBCT	Telephone and email	Individual	6	SBT	Weight; anxiety; depression; stress; psychological flexibility; binge eating; emotional eating; mindful eating	6
Daubenmier, 2011^67^,^87^	47	MBCT: 40.4 (8.0); No/min: 41.4 (6.7)	31.2 (4.8)	47 (100)	MBCT	Face to face	Group	4	No/min	Weight; anxiety; stress; disinhibition; emotional eating; dietary restraint;	4
Daubenmier, 2016^68^,^88^	194	MBCT: 47.2 (13.0); SBT: 47.8 (12.4)	MBCT: 35.4 (3.5); SBT: 35.6 (3.8)	MBCT: 79 (79); SBT: 81 (86)	MBCT	Face to face	Group	5½	SBT	Weight; DBP; SBP; fasting glucose; HbA1c; HDL; LDL; TG; TG HDL ratio; waist circumference	3, 6, 12, 18
Davis, 2008^35^	71	45.1 (8.3)	32.9 (3.7)	63 (89)	MBCT	Face to face	Group	6	SBT, SBT + RE	Weight; mindfulness; disinhibition; emotional eating; dietary restraint; hunger; body dissatisfaction	3, 6
Goldbacher, 2016^31^	79	45.6 (10.5)	36.2 (4.1)	75 (95)	MBCT	Face to face	Group	5	SBT	Weight; emotional eating	5
Kristeller, 2014^40^	150	46.6	40.3	132 (88)	MBCT	Face to face	Group	5¼	SBT, No/min	BMI; depression; binge eating; disinhibition; dietary restraint; hunger	6, 9
Lee,2017^36^	53	47.7 (11.3)	34.5 (4.8)	48 (91)	MBCT	Face to face	Group	3	SBT	Weight; stress; mindfulness; mindful eating; DBP; SBP; waist circumference	3, 6, 9
McKee, 2014^42^	60	37.6 (13.5)^c^	32.6 (4.9)^c^	40 (72)^c^	MBCT	Face to face and email	Group and individual	2	SBT	Weight; mindfulness; waist circumference	2, 3
Miller, 2012^69^,^89^	68	MBCT: 53.9 (8.2);SBT: 54.0 (7.0)^c^	MBCT: 36.2 (1.2); SBT: 36.1 (1.2)^c^	33 (63.5)^c^	MBCT	Face to face	Group	6	SBT	Weight; anxiety; depression; mindfulness; disinhibition; dietary restraint; hunger; fasting glucose; HbA1c; waist circumference	3, 4, 6
Palmeira, 2017^37^,^70^	73	MBCT: 42.0 (8.8) SBT: 42.7 (8.4)	MBCT: 34.8 (5.26) SBT: 33.7 (4.8)	73 (100)	MBCT	Face to face	Group	3½	SBT	BMI; QoL; mindfulness; disinhibition; emotional eating; total cholesterol; waist circumference	3.5
Raja‐Khan, 2017^32^,^61^	86	44.5 (12.5)	38.9 (8.7)	86 (100)	MBCT	Face to face	Group	2	SBT	Weight; anxiety; depression; stress; mindfulness; DBP; SBP; fasting glucose; HbA1c; LDL	2, 4
Smith, 2018^45^	40	MBCT: 58.6 (4.7); SBT: 58.6 (5.2)^c^	MBCT: 34.7 (4.3); SBT: 38.2 (7.1)^c^	36 (100)^c^	MBCT	Face to face	Group	12	SBT	Weight; binge eating	1½, 4, 9, 12
Spadaro, 2017^46^,^90^	49	45.2 (8.2)^c^	32.5 (3.7)^c^	40 (87)^c^	MBCT	Face to face	Group	6	SBT	Weight; mindfulness; disinhibition; dietary restraint; hunger	3, 6
Butryn, 2017^54^,^91^	283	ACT: 53.2 (9.4); SBT: 53.0 (9.3); BT + E: 53.4 (10.3)	ACT: 35.2 (4.6); SBT: 35.0 (5.2); BT + E: 35.4 (5.2)	ACT: 84 (82); SBT: 67 (76); BT + E: 72 (77)	ACT	Face to face	Group	12	SBT, BT + E	Weight	6, 12
Fletcher, 2011^30^	72	ACT: 53.1 (11.1); SBT: 52.1 (12.6)	ACT: 36.2 (0.6); SBT: 34.7 (0.6)	60 (83)	ACT	Face to face	Group	1 day	SBT	Weight; anxiety; depression; stress; psychological flexibility; DBP; SBP	1 week (not weight/BMI), 3
Forman, 2013^71^	128	45.7 (12.8)	34.1 (3.6)	NR	ACT	Face to face	Group	10	SBT	Weight	2½, 5, 10,12
Forman, 2016^72^,^92^	190	51.6 (10.1)	36.9 (5.8)	156 (82)	ACT	Face to face	Group	12	SBT	Weight	6, 12
Lillis, 2016 62,73,93	162	50.2 (10.9)	37.6 (5.3)	138 (85)	ACT	Face to face	Group	12	SBT	Weight; disinhibition	6, 12, 18, 24
Sairanen, 2017^44^, ^60^,^63^,^94^	219	49.5 (7.4)	31.3 (2.9)	185 (85)	ACT	Face to face Mobile	Group Individual	2	No/min	Weight; mindfulness; psychological flexibility; disinhibition; emotional eating; dietary restraint; intuitive eating	2½, 9
Loader, 2013^41^	36	45.4 (9.5)	46.7 (0.3)	25 (69)	CFT	Face to face and telephone	Group and individual	6	SBT	BMI; disinhibition; emotional eating; dietary restraint	6, 9
Adler, 2008^66^	17	49.4 (11.4)	37.7 (10.1)	15 (88)	DBT + O	Face to face and website	Group and individual	3	SBT + O		3, 4.5
*Preintervention to post‐intervention studies*											
Braun, 2012^28^	37	Range: 32‐65	NR	NR	MBCT	Face to face	Group	5 days	N/A	Weight; mindfulness; stress	5 days, 3, 12^d^
Braun, 2016^65^	S1: 22; S2: 21	S1: 48.2 (14.3); S2: 49.4 (10.7)	S1: 30.8 (4.2); S2: 35.5 (6.8)	S1: 22 (100); S2: 21 (100)	MBCT	Face to face	Group	2½	N/A	Weight; emotional eating; mindful eating	2.5, 5½
Chung, 2016^38^	26	50.1 (9.0)^c^	35.1 (4.0)^c^	22 (100)^c^	MBCT	Face to face and telephone	Group and individual	6	N/A	Weight; mindful eating	3¼, 6
Dalen, 2010^29^	10	44 (8.7)	36.9 (6.2)	7 (70)	MBCT	Face to face	Group	1½	N/A	Weight; anxiety; binge eating; depression; dietary restraint; disinhibition; stress	1½, 3
Hamel 2010^64^	10	50.4 (13.2)	29.1 (3.2)	9 (90)	MBCT	Face to face	Group	NR	N/A	BMI; emotional eating; hunger; mindful eating; QoL	3.25
Hanson 2019^77^	53	45.6 (11.3)	48.5 (9.2)	16 (30.2)	MBCT	Face to face	Group	2	SBT	Weight; emotional eating	½, 1, 1½, 2, 8
Lundgren, 2003^78^	33	44.8 (9.0)^c^	31.1 (3.6)^c^	16 (84)^c^	MBCT	Face to face	Group (unclear)	5	N/A	Weight; QoL	5
Andalcio 2018^76^	23	NR	39.9	21 (91.3)	ACT	Face to face and telephone	Individual	4	N/A	Weight; waist circumference	2, 4
Boucher, 2016^34^	40	44.8 (3.1)	32.9 (6.0)	40 (100)	ACT	Website	Individual	3½	N/A	BMI; intuitive eating	3½, 6.5
Bradley, 2017^79^,^95^	20	54.3 (12.1)	NR	17 (85)	ACT	Website and telephone	Individual	2½	N/A	Weight	1¼, 2½, 5½
Forman, 2009^80^	29	43.7 (9.8)	35.8 (5.4)	29 (100)	ACT	Face to face	Group	3	N/A	Weight; dietary restraint; disinhibition; emotional eating; mindfulness; QoL	3, 6
Niemeier, 2012^75^	21	52.2 (7.6)	32.8 (3.4)	19 (91)	ACT	Face to face	Group	6	N/A	Weight; dietary restraint; disinhibition; hunger	6, 9
Gallé,2017^39^	153	DBT:34 (3.8) IIT: 33 (4.2) SBT: 32 (5.1)	45.8 (6.4)	153 (100)	DBT	Face to face	Group and individual	12	IIT, SBT	Weight	12
Roosen, 2012^43^	35	39.2 (11.0)	35.4 (2.6)	30 (86)	DBT	Face to face	Group and individual	5	N/A	BMI; depression; dietary restraint; disinhibition; emotional eating	5, 11

Abbreviations: ACT, acceptance and commitment therapy; BMI, body mass index; BT + E, behaviour therapy with environmental change; CFT, compassion‐focused therapy; DBT, dialectical behavioural therapy; DBT + O, DBT + Orlistat; IIT, interpersonal individual treatment; MBCT, mindfulness‐based cognitive behavioural therapy; No/min, no/minimal intervention; NR, not reported; QoL, quality of life; RE, resistance exercise; S1, Study 1; S2, Study 2; SBT, standard behavioural treatment; SBT + O, SBT + Orlistat.

a Mean (SD) or range.

b Time since randomization/baseline.

c Among completers/participants included in analysis.

d 6 and 9 months recorded but not reported in the article.

#### 3.1.1 Participant characteristics

Studies included 2726 participants and the sample size ranged from 10^29^, ^64^ to 283.^54^ Seventy‐five percent of participants were female (n = 2035/2726), with 12 studies^28^, ^32^, ^33^, ^34^, ^38^, ^45^, ^64^, ^65^, ^67^, ^70^, ^80^ focusing exclusively on females. Mean age was 46 years (ranged from 21^33^ to 58 years^45^), and mean BMI was 35.6 kg/m^2^ (Table 1 and Table S2a,b).

#### 3.1.2 Intervention characteristics

Twenty‐two studies evaluated MBCT,^28^, ^29^, ^31^, ^32^, ^33^, ^35^, ^36^, ^38^, ^40^, ^42^, ^45^, ^46^, ^64^, ^65^, ^67^, ^68^, ^69^, ^70^, ^74^, ^77^, ^78^ eleven evaluated ACT‐based interventions,^30^, ^34^, ^44^, ^54^, ^71^, ^72^, ^73^, ^75^, ^76^, ^79^, ^80^ three evaluated DBT 39, 43, 66 (one^66^ in combination with pharmacotherapy), and one evaluated CFT.^41^ Twenty‐seven studies^28^, ^29^, ^30^, ^31^, ^32^, ^33^, ^35^, ^36^, ^40^, ^44^, ^45^, ^46^, ^64^, ^65^, ^67^, ^68^, ^69^, ^70^, ^71^, ^72^, ^73^, ^75^, ^77^, ^78^, ^80^, ^81^ used primarily face‐to‐face, group‐format delivery. One study^43^ had an initial individual face‐to‐face session before delivery of group sessions. Five other studies^38^, ^39^, ^41^, ^42^, ^66^ used face‐to‐face group sessions along with another mode: emails,^42^ telephone calls,^39^, ^41^ individual diet counselling^38^ and a website for pharmacology support.^66^ One study^76^ used individual face‐to‐face lifestyle counselling and telephone delivery. Three delivered interventions on an individual, remote basis using email^74^ and online website,^34^, ^79^ two of these included telephone support.^74^, ^79^ One study^44^ had two intervention arms with the same content delivered face to face or through mobile telephone. Most interventions include home‐based skills practice between sessions.

Intervention duration varied, with two lasting less than a week,^28^, ^30^ twelve studies between 1 and 3 months,^29^, ^32^, ^33^, ^36^, ^42^, ^44^, ^65^, ^66^, ^77^, ^79^, ^80^ and nine studies^31^, ^34^, ^40^, ^43^, ^67^, ^68^, ^70^, ^76^, ^78^ between 3½ and 5½ months in length. Thirteen studies lasted for 6 months or more, with five of these being 12 months in length.^39^, ^45^, ^54^, ^72^, ^73^ Hamel et al^64^ did not report intervention length.

All studies were delivered on a weekly or alternating weekly basis, apart from two: a one‐off 1‐day workshop and 5‐day residential retreat.^28^, ^30^ Several interventions had an “active phase,” then an extended period with less regular sessions or telephone follow‐up.^38^, ^40^, ^45^, ^54^, ^68^, ^69^, ^70^, ^71^, ^72^, ^73^ Most interventions were implemented at a university,^30^, ^35^, ^36^, ^40^, ^42^, ^45^, ^46^, ^54^, ^66^, ^68^ with other venues including primary care units and hospitals,^41^, ^70^, ^76^, ^77^ yoga retreat centres,^28^, ^65^ a community and oncology practice,^38^ YMCA,^29^ and participants' place of employment^80^ (Table 1 and Table S3a,b).

### 3.2 Risk of bias

Of the RCTs, the risk of bias was rated as 'high' in four,^36^, ^41^, ^42^, ^74^ 'some concern' in eleven,^30^, ^31^, ^33^, ^35^, ^40^, ^45^, ^46^, ^66^, ^69^, ^70^, ^71^ and 'low' in seven studies^32^, ^44^, ^54^, ^67^, ^68^, ^72^, ^73^ (Table S4a). Of the 15 non‐RCTs, the risk of bias was rated as 'serious' in nine,^28^, ^38^, ^39^, ^64^, ^65^, ^78^, ^79^, ^80^ and 'moderate' in six^29^, ^34^, ^43^, ^75^, ^76^, ^77^ studies (Table S4b). The quality of the evidence was different for different comparisons, dependent on studies included. For the comparison between 3wCBT and no/minimal intervention at post‐intervention, the quality of evidence was 'high' (three studies);^40^, ^44^, ^67^ for the comparison between 3wCBT and SBT, the quality of evidence was 'moderate' at post‐intervention (nineteen studies)^30^, ^31^, ^32^, ^33^, ^35^, ^36^, ^40^, ^41^, ^42^, ^45^, ^46^, ^54^, ^68^, ^69^, ^70^, ^71^, ^72^, ^73^, ^74^ and 'high' at 12 months (five studies),^45^, ^54^, ^68^, ^72^ 18 months (three studies),^68^, ^71^, ^73^ 24 months (two studies),^72^, ^73^ and 36 months (one study,^72^ direct evidence only) from baseline. Details of study quality for all comparisons are reported Table S5a‐c.

### 3.3 Intervention effects on body weight or BMI

Twenty‐five studies^28^, ^29^, ^30^, ^31^, ^32^, ^33^, ^35^, ^36^, ^38^, ^42^, ^44^, ^45^, ^46^, ^54^, ^65^, ^67^, ^68^, ^69^, ^73^, ^74^, ^75^, ^76^, ^77^, ^78^ reported an absolute weight change (kg or lb), four studies^71^, ^72^, ^79^, ^80^ reported percent change from baseline weight, and six studies^34^, ^40^, ^41^, ^43^, ^64^, ^70^ reported BMI change.

#### 3.3.1 Pooled estimates of intervention‐specific effects from all study types

Standardized mean change in weight or BMI for 3wCBT was −0.84 (95% CI: −1.06, −0.62; N = 35; I^2^ = 93%) from baseline to post‐intervention (equating an absolute weight change of 5.5 kg). Weight change by types of 3wCBT at different time points is reported in Table S6. There was a pattern of weight loss (relative to baseline) for DBT up to 12 months, for MBCT up to 18 months, and for ACT up to 36 months. However, there was high heterogeneity and few studies at later time points. There was no evidence of weight loss following CFT, but this was based on one poor‐quality study^41^ at a single 3 month follow‐up from baseline.

#### 3.3.2 Intervention comparisons: Direct evidence

**Figure 2:**
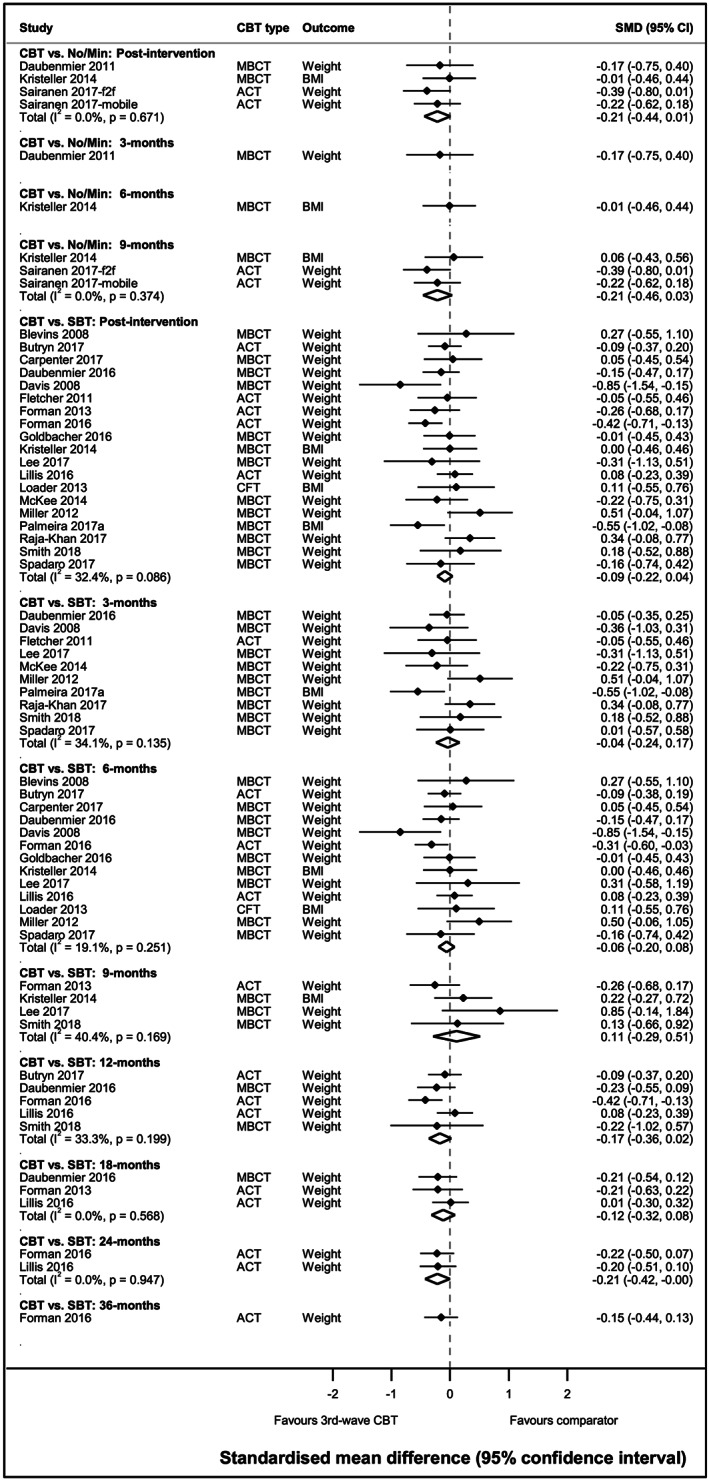
Weight change comparing third‐wave CBT and no/minimal or standard behavioural treatment from random‐effects pairwise meta‐analysis. Time points are months since baseline unless otherwise specified. CBT, cognitive behaviour therapy; MBCT, mindfulness‐based cognitive behaviour therapy; No/min, no/minimal intervention; SBT, standard behavioural treatment; SMD, standardized mean difference

Figure 2 summarizes the results of the pairwise random‐effects meta‐analysis, and Table S5a presents the quality of evidence for all direct comparisons using GRADE.^27^ Most individual studies were small and did not find evidence of a difference between interventions. However, when findings were meta‐analysed, there was high‐quality evidence to suggest greater weight loss for 3wCBT compared with no/minimal intervention at post‐intervention and 9 month follow‐up from baseline. There was moderate‐quality evidence based on 19 studies,^30^, ^31^, ^32^, ^33^, ^35^, ^36^, ^40^, ^41^, ^42^, ^45^, ^46^, ^54^, ^68^, ^69^, ^70^, ^71^, ^72^, ^73^, ^74^ suggesting that 3wCBT had greater weight loss than SBT at post‐intervention, and high‐quality evidence suggesting that 3wCBT had greater weight loss than SBT at 12 months (five studies^45^, ^54^, ^68^, ^72^, ^73^) and 24 months (two studies^72^, ^73^). Only ACT interventions provided data for the 24 month comparison. Estimates at 6 and 18 months also suggested greater weight loss for 3wCBT versus SBT, but there was no evidence of a difference between the two groups at 3 and 9 months.

#### 3.3.3 Sensitivity analysis

In the influence plot analysis, removal of one study at a time did not have any effect on the overall effects estimates from the pairwise meta‐analysis of weight change.

#### 3.3.4 Intervention comparisons: Indirect and mixed evidence

**Figure 3:**
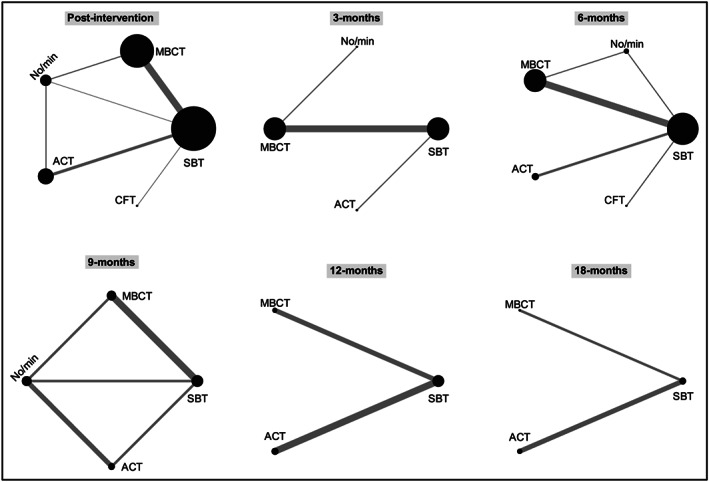
Network of interventions at different follow‐up from baseline time points. Nodes are weighted by the number of studies involved in each intervention while the edges are weighted by the number of studies involved in each comparison. Time points are months since baseline unless otherwise specified. ACT, acceptance and commitment therapy; CFT, compassion‐focused therapy; MBCT, mindfulness‐based cognitive behaviour therapy; No/min, no/minimal intervention; SBT, standard behavioural treatment

**Figure 4:**
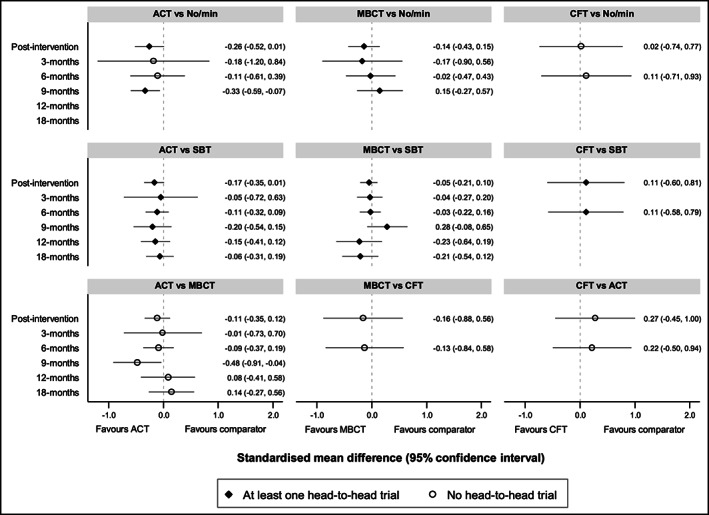
Summary of weight change from network meta‐analysis at different follow‐up from baseline time points. Time points are months since baseline unless otherwise specified. ACT, acceptance and commitment therapy; CFT, compassion‐focused therapy; MBCT, mindfulness‐based cognitive behaviour therapy; No/min, no/minimal intervention; SBT, standard behavioural treatment

Network meta‐analysis was conducted up to 18 months post‐baseline, as only a single pairwise comparison (ACT vs. SBT) was reported at 24 and 36 months. Intervention networks at each time point are summarized in Figure 3. Estimates from the network meta‐analysis are summarized in Figure 4. ACT produced greater weight loss than no/minimal intervention at post‐intervention and 9 months; comparisons at 3 and 6 months, however, did not provide evidence of superior effectiveness of ACT. Comparisons between ACT and SBT suggested greater weight loss for ACT post‐intervention. There was no evidence of a difference at other time points. Compared with MBCT, ACT had greater weight loss at 9 months; comparisons at other time points did not show evidence of a difference.

Comparisons between no/minimal intervention and MBCT did not provide evidence of a difference at any time point, and there was no consistent pattern of effects. Comparisons between MBCT and SBT suggested that SBT was more effective at 9 months, but estimates at 12 and 18 months suggested that MBCT was favoured. When CFT was compared with the other interventions, CIs were wide with no comparisons favouring CFT.

When interventions were relatively ranked, ACT was the best intervention post‐intervention and at 3, 6, and 9 months post‐baseline. MBCT was the best ranking intervention at 12 and 18 months post‐baseline; however, this was based on only five studies (two MBCT) and three studies (one MBCT), respectively (Figure S1).

In terms of absolute weight change, for example, the SMD in weight between 3wCBT and SBT equates to a difference of 0.6 kg post‐intervention and 1.4 kg at 24‐month follow‐up from baseline.

### 3.4 Interventions effects on secondary outcomes

**Figure 5:**
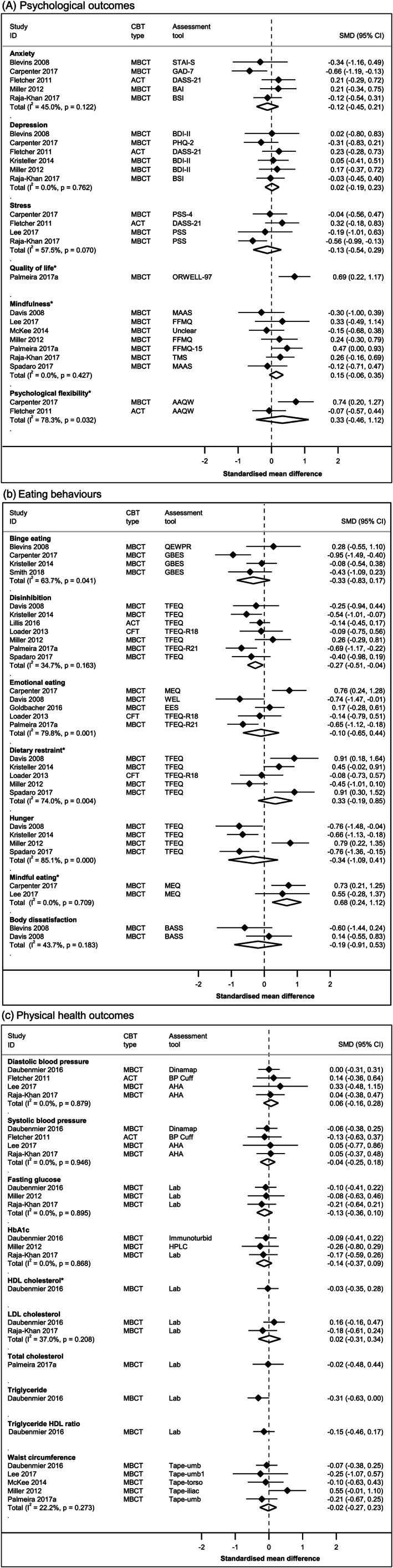
Changes in secondary outcomes comparing third‐wave cognitive behaviour therapy and standard behavioural treatment at earliest time point post‐intervention using random‐effects pairwise meta‐analysis. A, Psychological outcomes. B, Eating behaviours. C, Physical health outcomes. For variables with asterisks (*), estimates to the right of the dotted line indicate a desired change in favour of third‐wave CBTs; for all other variables, it is to the left of the dotted line. ACT, acceptance and commitment therapy; CBT, cognitive behaviour therapy; MBCT, mindfulness‐based cognitive behaviour therapy; SMD, standardized mean difference

Pooled arm‐specific estimates (standardized mean change) of the effect of 3wCBT (combined) on secondary outcomes are presented in Figure S2. Pairwise estimates (SMD) from RCTs comparing 3wCBT and no/minimal intervention are presented in Figure S3; those comparing 3wCBT and SBT are presented in Figures 5A, 5B, and 5C.

#### 3.4.1 Psychological outcomes

Pooled arm‐specific estimates showed a reduction in anxiety (N = 7),^29^, ^30^, ^32^, ^33^, ^67^, ^69^, ^74^ depression (N = 9),^29^, ^30^, ^32^, ^33^, ^40^, ^43^, ^69^, ^74^, ^78^ and stress (N = 8)^28^, ^29^, ^30^, ^32^, ^36^, ^67^, ^74^, ^78^ following 3wCBT. When compared with no/minimal intervention, estimates suggested greater reductions in anxiety for 3wCBT (N = 1),^67^ a greater reduction in depression for 3wCBT (N= 1),^40^ but found no evidence of a difference in stress (N = 1).^67^ Pairwise comparisons found no evidence of differences between 3wCBT and SBT for anxiety (N = 5),^30^, ^32^, ^33^, ^69^, ^74^ depression (N = 6),^30^, ^32^, ^33^, ^40^, ^69^, ^74^ or stress (N = 4).^30^, ^32^, ^36^, ^74^

Pooled arm‐specific estimates (N = 4)^64^, ^70^, ^78^, ^80^ suggested an increase in quality of life following 3wCBT. One study^70^ reported a greater increase in quality of life in 3wCBT versus SBT.

Pooled arm‐specific estimates (N = 9)^32^, ^35^, ^36^, ^42^, ^44^, ^46^, ^69^, ^70^, ^80^ showed an increase in mindfulness with 3wCBT. The estimate from one study^44^ suggested a greater increase in mindfulness for 3wCBTs versus no/minimal intervention. Pairwise estimates (N = 7)^32^, ^35^, ^36^, ^42^, ^46^, ^69^, ^70^ suggested a greater increase in mindfulness in 3wCBT versus SBT.

Pooled arm‐specific estimates (N = 5)^30^, ^34^, ^44^, ^74^, ^75^ showed that psychological flexibility increased after 3wCBT. One study^44^ showed a greater increase in psychological flexibility for 3wCBT versus no/minimal intervention. Pairwise estimates (N = 2)^30^, ^74^ found no evidence of a difference between 3wCBT and SBT.

#### 3.4.2 Eating behaviour

Pooled arm‐specific estimates showed a decrease in binge eating (N = 6),^29^, ^33^, ^40^, ^45^, ^74^, ^78^ disinhibition (N = 14),^29^, ^35^, ^40^, ^41^, ^43^, ^44^, ^46^, ^67^, ^69^, ^70^, ^73^, ^75^, ^78^, ^80^ and hunger (N = 7)^29^, ^35^, ^40^, ^46^, ^69^, ^75^, ^78^ and suggested a decrease in emotional eating (N = 13)^31^, ^35^, ^41^, ^43^, ^44^, ^64^, ^65^, ^67^, ^70^, ^74^, ^77^, ^80^ following 3wCBT. Compared with no/minimal intervention, three studies^40^, ^44^, ^67^ showed a greater decrease in disinhibition favouring 3wCBT, one study^40^ reported a greater decrease in binge eating and hunger favouring 3wCBT, and two studies^44^, ^67^ showed a greater decrease in emotional eating favouring 3wCBT. Pairwise estimates comparing 3wCBT and SBT found a greater decrease in disinhibition for 3wCBT (N = 7),^35^, ^40^, ^41^, ^46^, ^69^, ^70^, ^73^ but no evidence of a difference between the groups in binge eating (N = 4),^33^, ^40^, ^45^, ^74^ hunger (N = 4),^35^, ^40^, ^46^, ^69^ or emotional eating (N = 5).^31^, ^35^, ^41^, ^70^, ^74^ Pooled arm‐specific estimates showed an increase in dietary restraint (N = 12),^29^, ^35^, ^40^, ^41^, ^43^, ^44^, ^46^, ^67^, ^69^, ^75^, ^78^, ^80^ intuitive eating (N = 2),^34^, ^44^ and mindful eating (N = 6)^36^, ^38^, ^64^, ^65^, ^74^ following 3wCBT. Pairwise estimates comparing 3wCBT and no/minimal intervention showed a greater increase in dietary restraint (N = 3)^40^, ^44^, ^67^ and a greater increase in intuitive eating (N = 1)^44^ in the 3wCBT group. Pairwise estimates comparing 3wCBT and SBT found a greater increase in mindful eating for 3wCBT (N = 2)^36^, ^74^ but no evidence of a difference in dietary restraint (N = 5).^35^, ^40^, ^41^, ^46^, ^69^ No studies compared intuitive eating in 3wCBT versus SBT.

Pooled estimates (N = 6)^33^, ^35^, ^43^, ^65^, ^78^ showed no evidence of a change in body dissatisfaction following 3wCBT; pairwise comparisons showed no evidence of a difference between 3wCBT and SBT (N = 2).^33^, ^35^

#### 3.4.3 Physical health outcomes

Pooled arm‐specific estimates (N = 4)^30^, ^32^, ^36^, ^68^ suggested a reduction in diastolic blood pressure and systolic blood pressure following 3wCBT, but pairwise estimates showed no evidence of differences between 3wCBT and SBT. Pooled arm‐specific estimates (N = 3)^32^, ^68^, ^69^ suggested a reduction in fasting glucose and HbA_1c_ following 3wCBT, and pairwise estimates suggested greater reductions in fasting glucose and HbA_1c_ for 3wCBT versus SBT. There was no evidence of changes in high‐density lipoprotein cholesterol (N = 1),^68^ low‐density lipoprotein cholesterol (N = 2),^32^, ^68^ or total cholesterol (N = 1) following 3wCBT,^70^ and no evidence of differences between 3wCBT and SBT in any of these outcomes. One study^68^ reported a decrease in triglyceride and triglyceride‐to‐HDL ratio following 3wCBT and a greater decrease of triglyceride in 3wCBT compared with SBT with no evidence of a change for triglyceride‐to‐HDL ratio between 3wCBT and SBT. Pooled arm‐specific estimates (N = 6)^36^, ^42^, ^68^, ^69^, ^70^, ^76^ showed a decrease in waist circumference following 3wCBT, but pairwise comparisons found no evidence of a difference between 3wCBT and SBT.

### 3.5 Meta‐regression of intervention characteristics

A sufficient number of studies for meta‐regression (N ≥ 10) were only available at post‐intervention and at 3‐ and 6‐ months since baseline for 3wCBTs versus SBT. Prespecified study and intervention characteristics were examined in the meta‐regression at these time points including number of sessions, duration of intervention, method of delivery, and risk of bias (Table S7), and none were found to have any impact on the effect estimates on weight or BMI reported in the pairwise meta‐analysis. There were too few studies in each stratum to analyse the potential effects of comorbidities (eg, diabetes). Due to the small number of studies, subgroup analysis was not conducted.

### 3.6 Intervention adherence

There was substantial heterogeneity and poor reporting of attendance and adherence outcomes, limiting our ability to conduct any meaningful quantitative analysis (Table S8). Only 22 studies reported any attendance information, but, for all these studies, attendance was at least 60% at group sessions overall, and eight^31^, ^35^, ^42^, ^46^, ^54^, ^69^, ^71^, ^72^ out of 11 RCTs reporting attendance information for each group had a 3wCBT group with higher attendance than the control arm. Information provided on adherence included minutes of home meditation practice, number of mindful meals per week, food and exercise diaries, and completion of online modules. Generally, within each study, there seemed to be a spread of engagement in the home practice aspect of interventions. This also varied with interventions delivered via internet: one study^44^ found a 91% median completion of all modules, and another^34^ found a mean of 32%.

## 4 DISCUSSION

This comprehensive systematic review and network meta‐analysis found high‐quality evidence suggesting that 3wCBT results in greater weight loss than no/minimal intervention. Importantly, it also found moderate‐quality evidence that suggests that 3wCBT results in greater weight loss than SBT at post‐intervention and high‐quality evidence from a small number of studies indicating that 3wCBT results in greater weight loss than SBT at longer term follow‐up. However, it is important to note that 3wCBTs did not consistently outperform other interventions across shorter follow‐up times (eg, 3‐ and 9‐ months) and that differences in weight change between 3wCBT and SBT were small (approximately 0.6 kg difference post‐intervention and 1.4 kg difference at 24 ‐months). Future research is needed to establish the clinical significance of these small differences in weight change.

The finding that 3wCBT is potentially more effective than SBT contrasts with the report by Roche et al^14^ that acceptance‐ and mindfulness‐based interventions were only more effective than waitlist control arms. Conversely, estimates for the difference in weight loss between CBT and no/minimal intervention in our analysis are slightly smaller. This may be because Roche et al^14^ merged short‐ and long‐term follow‐up data and included participants with a BMI less than 25kg/m^2^, either of which may be associated with larger effects. Comparisons with other reviews that purport to have compared 3wCBT with other approaches are more challenging because of less appropriate statistical approaches. Both Carrière et al^15^ and Rogers et al^13^ combined the estimates from both RCTs and single‐arm pre‐intervention to post‐intervention studies. While the analytic methods used in Carrière et al^15^ is unclear, Rogers et al^13^ used only the post‐intervention estimates for the RCTs (which ignores baseline differences between groups that may be influential in smaller studies),^82^ and effect estimates were weighted by sample size, not SD. Consequently, the reported study‐specific estimates are different between Rogers et al^13^ and Roche et al^14^ even though both label the effect as Hedges' g.^15^, ^59^

Our statistical approach was more rigorous. In the pairwise meta‐analysis, we only included RCTs, we applied a consistent definition of 'change' estimate as pre‐intervention minus post‐intervention estimates, we used appropriate methods for pairwise comparisons, and we further separated the effectiveness against no/minimal control or SBTs. We reported the effect estimates by follow‐up times to have more insights of the short‐term and longer term effects, and we restricted our analytic population to those with a BMI greater than or equal to 25 kg/m^2^ as this is more relevant from a clinical and policy perspective.

To our knowledge, this is also the first review to report on the comparative effectiveness of individual 3wCBT types, which have never been tested in head‐to‐head trials. Network meta‐analysis found that ACT‐based interventions had the most consistent evidence of effectiveness indicating greater weight loss compared with SBT at post‐intervention and 12‐ and 24‐month follow‐up from baseline; comparisons at other time points or with other 3wCBT types either appeared to favour ACT or did not show evidence of a difference. ACT was ranked as the best intervention up to 12 months and was the only 3wCBT to have outcomes at 24 and 36 months. Network estimates suggested that MBCT resulted in greater weight loss than SBT at 12 and 18 months, but favoured SBT at 9 months, and there was no evidence that MBCT was more effective than no/minimal intervention. This suggests that we should interpret the finding that MBCT was the highest ranking intervention at 12 and 18 months with some caution. Only four studies evaluated a 3wCBT approach other than acceptance or mindfulness and these were of low quality and short follow‐up. Although we identified three studies using DBT, all used non‐randomized pre‐intervention to post‐intervention design and one was combined with pharmacotherapy; therefore, they were not included in the pairwise or network meta‐analysis, limiting conclusions on DBT effectiveness. CFT was found to have no evidence for weight loss; however, this finding is based upon one unpublished thesis,^41^ which was deemed to be of high risk of bias and of very low quality. To date, the evidence provides strongest support for the superiority of acceptance‐based interventions. It is possible that the superiority of the acceptance‐based programmes in this context is due to its focus on values and willingness to reduce experiential avoidance. However, more research is needed to confirm these differences and identify the mechanisms of action.

Changes in secondary outcomes were generally in the desired direction. Following 3wCBT, there was evidence suggestive of reductions in depression, anxiety, and stress and increases in quality of life, mindfulness, and psychological flexibility. There were similar reductions in binge eating, dietary disinhibition, hunger, and emotional eating and increases in dietary restraint, intuitive eating, and mindful eating following 3wCBT. This is consistent with previous reviews that have reported on these outcomes.^13^, ^15^, ^16^ Pairwise comparisons suggest that most improvements in these outcomes were greater than for no/minimal intervention. Focusing on comparisons of 3wCBT versus SBT, pairwise comparisons showed that reductions in disinhibition and increases in mindful eating were greater for 3wCBT but no differences in other psychological factors. These could represent important mechanisms by which 3wCBT has a greater effect on weight control than SBT and warrant further investigation. Few studies reported changes in blood pressure, glycaemia, lipid profile, and waist circumference, and pairwise comparisons only suggested greater reductions in glycaemia.

Meta‐regression did not identify any specific intervention characteristics (eg, duration, mode of delivery, number of sessions) that were more effective than others. This may have been due to the small number of studies. Similar to traditional behavioural weight management programmes, the majority of interventions were delivered in a group face‐to‐face format. Such delivery has often been found to be effective in weight loss,^83^ with the group providing peer support and regular encouragement, particularly for those experiencing social isolation. However, closed‐group programmes led by clinical psychologists can be logistically difficult and costly to deliver, particularly in the context of national health services providing free or discounted health care. Increasingly, standard behavioural weight management interventions are moving to more scalable methods of delivery to increase reach and reduce cost. Only four interventions^34^, ^44^, ^74^, ^79^ in our review used remote delivery through internet or mobile phone. Similarly, a review of online mindful eating interventions^18^ found only two studies focusing on weight loss, and none were delivered through mobile telephone, highlighting a dearth of investigation into this research area. Greater consideration may need to be given to the use of digital technology to facilitate intervention delivery, with its 24‐hour accessibility, improved scalability, and increased reach.^84^

## 5 STRENGTHS AND LIMITATIONS

The study of 3wCBT for weight management is in its relative infancy, with the earliest included RCTs from 2008^33^ (MBCT) and 2011^30^ (ACT). While a number of RCTs have been conducted, most have had small samples and short follow‐up and few provide high‐quality evidence. Only three RCTs reported outcomes beyond 12 months despite 3wCBT being hypothesized to have particular benefit for long‐term outcomes. However, these studies with longer term follow‐up had low risk of bias and provided high‐quality evidence. The small number of studies limited our network meta‐analysis to up to 18 months post‐baseline and meant that there were insufficient studies to conduct meaningful meta‐regression on the most effective intervention components and characteristics beyond 6 months. Many studies also had very small sample sizes. The small number of studies and small sample sizes meant that many of our estimates had wide CIs, thereby limiting the power to detect a difference. Many of the studies included in this review had high or serious risk of bias. However, it should be noted that we used a stringent assessment tool, and heterogeneity for many outcomes was low. For some studies, this may also reflect the slowness of the obesity field to adopt standards for trial reporting (eg, CONSORT),^23^ rather than the quality of the research itself. It is also important to note that the studies with longer term follow‐up (ie, 12 and 24 months from baseline) were of high quality, based upon the GRADE assessment tool,^27^ so we can have greater confidence in the findings at these time points.

There was heterogeneity in the content of 3wCBT programmes, with a combination of standardized, modified, and novel programmes that varied in length and practice time. Some studies evaluated interventions that used combinations of different third‐wave therapies, which may obscure potential differences between types of therapy. However, this is a reflection of how these interventions are used. In attempting to collate adherence and attendance data, we found a low number of studies reporting intervention fidelity information and substantial variability in reporting. 3wCBTs seem to have comparable attendance and attrition rates to standard behavioural programmes,^85^ suggesting that they are an acceptable and feasible option. Lack of information stopped us from conducting a meta‐regression to try to identify sources of heterogeneity in attendance or adherence.

Studies in our review had a high proportion of female participants; this is typical in weight loss programmes and mindfulness interventions.^86^ This limits generalizability of findings to males^83^ and warrants purposive recruitment of males to studies and weight loss programmes per se. Furthermore, several studies lacked detail of participant demographics such as ethnicity and socio‐economic status; more complete reporting would enable us to understand the extent of the generalizability of results.

There are several strengths to this review. RCTs and pre‐intervention to post‐intervention studies were included in our pooled estimates, but only RCTs were included in pairwise and network meta‐analysis to provide the estimates of the comparative effectiveness against a comparator. By conducting a network analysis, we could estimate comparisons between different types of 3wCBT that have not been directly compared, incorporating direct, indirect, and mixed evidence in our evaluations of the evidence. To maximize on relevant research, we included unpublished theses and contacted authors regarding abstracts in conference proceedings. Unlike previous reviews,^14^ we restricted our analytic population to those with a BMI ≥25 kg/m^2^ to make our results more relevant to health care policies that recommend weight management interventions for people with overweight/obesity.

## 6 CONCLUSIONS

This systematic review and network meta‐analysis found moderate‐quality evidence suggesting that 3wCBT results in a small increase in weight loss compared with SBT at post‐intervention. It found high‐quality evidence from a small number of studies suggesting that 3wCBT results in greater weight loss than SBT at 12 and 24 month follow‐up from baseline. Evidence specifically appears to support the use of acceptance‐based programmes. Larger, high‐quality trials are needed in this area to better understand who these interventions work for and how they work, so that we can target these interventions appropriately and identify the most crucial components and “active ingredients.” Future research should also consider how we deliver these interventions in a cost‐effective way that maximizes scalability while maintaining effectiveness.

## CONFLICTS OF INTEREST

AA is the chief investigator on two publically funded (MRC, NIHR) trials where the intervention is provided by WW (formerly Weight Watchers) at no cost outside the submitted work. AJH reports receiving personal fees from Slimming World, outside the submitted work. CAH reports education work and consultancy Oviva, Orexigen Therapeutics, Kastech, Ethicon, Mundipharma, Consilient Health, Nestle, and Novo Nordisk, outside the submitted work. ERL, NI, SB, and SJG have no conflicts of interest.

## Supporting information


**Table S1.** Medline search terms
**Table S2a.** Participant characteristics of included randomised controlled trials
**Table S2b.** Participant characteristics of included pre‐post studies
**Table S3a.** Intervention characteristics of included randomised controlled trials
**Table S3b.** Intervention characteristics of included pre‐post design studies
**Table S4a.** Risk of bias judgements for randomised controlled trials (RoB 2.0)
**Table S4b.** Risk of bias judgements for non‐randomised studies (ROBINS‐I)
**Table S5a.** GRADE assessment at different follow‐up time points (DIRECT EVIDENCE)
**Table S5b.** GRADE assessment at different follow‐up time points (INDIRECT EVIDENCE)
**Table S5c.** GRADE assessment at different follow‐up time points (OVERALL EVIDENCE)
**Table S6.** Pooled effects estimates of third‐wave cognitive behaviour therapies on weight change estimated from random‐effects meta‐analysis
**Table S7.** Meta‐regression analysis of the effects of third‐wave cognitive behaviour therapies on weight management compared to standard behavioural treatment
**Table S8a.** Attendance and adherence information of randomised controlled trials
**Table S8b.** Attendance and adherence information of pre‐post studies
**Figure S1.** Ranking plot showing the probability of each of the evaluated interventions of ranking the best to the worst intervention
**Figure S2.** Pooled effects estimates of third‐wave cognitive behaviour therapies on secondary outcomes estimated from random‐effects meta‐analysis at the earliest measurement post‐ intervention
**Figure S3.** Effects on secondary outcomes comparing third‐wave cognitive behaviour therapies and no/minimal intervention from random‐effects pairwise meta‐analysis at the earliest measurement post‐intervention
