# Supplementary material for: Third‐wave cognitive behaviour therapies for weight management: A systematic review and network meta‐analysis
Source: Obes Rev. 2020 Mar 17;21(7):e13013. doi: 10.1111/obr.13013 (PMC7379202; doi:10.1111/obr.13013)
Supplement: Supplementary file 1 — Table S1. Medline search terms Table S2a. Participant characteristics of included randomised controlled trials Table S2b. Participant characteristics of included pre‐post studies Table S3a. Intervention characteristics of included randomised controlled trials Table S3b. Intervention characteristics of included pre‐post design studies Table S4a. Risk of bias judgements for randomised controlled trials (RoB 2.0) Table S4b. Risk of bias judgements for non‐randomised studies (ROBINS‐I) Table S5a. GRADE assessment at different follow‐up time points (DIRECT EVIDENCE) Table S5b. GRADE assessment at different follow‐up time points (INDIRECT EVIDENCE) Table S5c. GRADE assessment at different follow‐up time points (OVERALL EVIDENCE) Table S6. Pooled effects estimates of third‐wave cognitive behaviour therapies on weight change estimated from random‐effects meta‐analysis Table S7. Meta‐regression analysis of the effects of third‐wave cognitive behaviour therapies on weight management compared to standard behavioural treatment Table S8a. Attendance and adherence information of randomised controlled trials Table S8b. Attendance and adherence information of pre‐post studies Figure S1. Ranking plot showing the probability of each of the evaluated interventions of ranking the best to the worst intervention Figure S2. Pooled effects estimates of third‐wave cognitive behaviour therapies on secondary outcomes estimated from random‐effects meta‐analysis at the earliest measurement post‐ intervention Figure S3. Effects on secondary outcomes comparing third‐wave cognitive behaviour therapies and no/minimal intervention from random‐effects pairwise meta‐analysis at the earliest measurement post‐intervention [file OBR-21-e13013-s001.docx]

**Third-wave cognitive behaviour therapies for weight management: a systematic review and network meta-analysis**

Emma R Lawlor^1*^, Nazrul Islam^1*^, Sarah Bates^2^, Simon J Griffin^1,3^, Andrew J Hill^4^, Carly A Hughes^5,6^, Stephen Sharp^1^, Amy L Ahern^1^

1. MRC Epidemiology Unit, University of Cambridge, Cambridge, United Kingdom
2. School of Health and Related Research, Faculty of Medicine, Dentistry and Health, University of Sheffield, Sheffield, United Kingdom
3. Primary Care Unit, Institute of Public Health, University of Cambridge, Cambridge, United Kingdom
4. Division of Psychological & Social Medicine, School of Medicine, University of Leeds, Leeds, United Kingdom
5. Fakenham Medical Practice, Norfolk, United Kingdom.
6. Norwich Medical School, University of East Anglia, Norwich, United Kingdom.

*Joint contribution.

Corresponding author: Dr Emma R Lawlor

Address: MRC Epidemiology Unit, University of Cambridge, School of Clinical Medicine, Box 285 Institute of Metabolic Science, Cambridge Biomedical Campus, Cambridge, CB2 0QQ, United Kingdom.

Email: emma.lawlor@mrc-epid.cam.ac.uk

Running title: Third-wave cognitive behaviour therapies for weight management

Keywords: Weight loss; Obesity; Network meta-analysis; Third-wave behavioural therapy

**Table S1; Medline search terms**

| 1 | exp Obesity/ |
| --- | --- |
| 2 | exp Overweight/ |
| 3 | exp Body Weight/ |
| 4 | exp Body Mass Index/ |
| 5 | exp Waist Circumference/ |
| 6 | exp Feeding Behavior/ |
| 7 | exp Body Weight Changes/ |
| 8 | exp Caloric Restriction/ |
| 9 | exp Weight Loss/ |
| 10 | obes*.mp. |
| 11 | (overweight or over-weight).mp. |
| 12 | (weight adj3 (body or chang* or loss* or maint* or manag* or control* or reduct*)).mp. |
| 13 | (food adj3 (intake or habit*)).mp. |
| 14 | (body mass index or bmi).mp. |
| 15 | body adj3 mass.mp. |
| 16 | (calori* adj3 (restrict* or restrain* or reduc*)).mp. |
| 17 | feeding adj3 behavio*.mp. |
| 18 | (diet* adj3 (restrict* or restrain* or reduc*)).mp. |
| 19 | (waist* adj3 circumferenc*).mp. |
| 20 | 1 or 2 or 3 or 4 or 5 or 6 or 7 or 8 or 9 or 10 or 11 or 12 or 13 or 14 or 15 or 16 or 17 or 18 |
| 21 | ((3rd or third) adj3 wave).mp. |
| 22 | (acceptance* adj3 (commit* or mind* or base* or focus* or intervention* or therap* or treat*)).mp. |
| 23 | exp Mindfulness/ |
| 24 | (mindful* or mind-ful*).mp. |
| 25 | (compassion* adj3 (mind* or base* or focus* or intervention* or therap* or treat*)).mp. |
| 26 | (behav* adj3 activation).mp. |
| 27 | ((meta-cognit* or metacognit*) adj3 (mind* or base* or focus* or intervention* or therap* or treat*)).mp. |
| 28 | (dialectic* adj3 (behavio* or mind* or base* or focus* or intervention* or therap* or treat*)).mp. |
| 29 | (schema* adj3 (mind* or base* or focus* or intervention* or therap* or treat*)).mp. |
| 30 | (function* adj3 analyt*).mp. |
| 31 | (relation* adj3 frame*).mp. |
| 32 | 20 or 21 or 22 or 23 or 24 or 25 or 26 or 27 or 28 or 29 or 30 |
| 33 | 20 and 32 |

Search terms 21 to 31 based upon Linardon et al.’s (2017)^[[1]](#footnote-1)^ search strategy. No database search restrictions were applied.

**Table S2a, Participant characteristics of included randomised controlled trials**

| 1st author (year) | Study population | Recruitment method | Inclusion criteria | Race/Ethnicity; N (%) | Co-morbidities | Education |
| --- | --- | --- | --- | --- | --- | --- |
| Studies with an intervention using MBCT | |  |  |  |  |  |
| Blevins, 2008 (33) | Female university students (18-25 yrs) with body mass index (BMI)>25 kg/m^2^ | Advertisements on university campus & student website | BMI 25-35 kg/m^2^; aged 18-25 years; Female | African American: 21 (51%); Caucasian: 13 (32%); Hispanic: 5 (12%); Asian American: 2 (5%) | NR | Senior year of college: 51%; Graduate: 10% |
| Carpenter, 2017 (74) | Adult enrolees of phone-based employer-sponsored behavioural weight loss programme with BMI 25-35 kg/m^2^ & high emotional eating scores | Enrolees in employer-sponsored weight loss programme (Weight Talk) | BMI 25-35 kg/m^2^; aged ≥18 years; high emotional eating scores; regular access to email & the internet | White non-Hispanic: 49 (65%); Black non-Hispanic: 20 (27%); Hispanic: 5 (7%); Asian: 1 (1%). | NR | High school or less: 5.3%; Some college: 37.3%; College graduate: 57.3% |
| Daubenmier, 2011 (67; 87) | Premenopausal adult women with BMI 25–40 kg/m^2^ (& weighed less than 300 lbs) who wanted to control the effects of stress on their eating behaviour | Media outlets & flyers posted in San Francisco Bay Area | BMI 25-40 kg/m^2^; weigh <300 lbs; pre-menopausal; No medical issues | White: 29 (62%); Hispanic/Latino: 7 (15%); Asian/Pacific Islander: 7 (15%); Other: 4 (9%) | NR | NR |
| Daubenmier, 2016 (68; 88) | Adults with BMI 30-45.9 kg/m^2^ with abdominal obesity | Fliers, newspaper advertisements, online postings & referrals at university clinics | BMI 30-45.9 kg/m^2^; aged ≥18 years; waist ≥102 cm (men) or ≥88 cm (women) | European: 115 (59%); African: 25 (13%); Asian/Pacific Islander: 19 (10%); Latina/Latino: 23 (12%); Other: 10 (5%) | NR | Bachelor’s degree:  *Intervention*:  69 (69.7%);  *Control*:  56 (59.6%) |
| Davis, 2008 (35) | Adults (18-55 years) with BMI 25-39.9 kg/m^2^ | Media outlets (e.g. local newspaper, newsletter, television & radio advertisements) | BMI 25.0-39.9 kg/m^2^; aged 18-55 years | African American: 18 (25%); Caucasian: 52 (73%); Other: 1 (1%) | NR | NR |
| Goldbacher, 2016 (31) | Adults with BMI 30-45 kg/m^2^ with high levels of emotional eating | Newspaper advertisements, flyers & physician referrals between Jan & Aug 2010 | BMI 30-45 kg/m^2^; aged 21-65 years; top tertile on any subscale of the Emotional Eating Scale | Black/African American: 65 (82%); White: 10 (13%); Mixed/Other: 4 (5%) | NR | Highest year in school completed: mean: 14.5 (SD=1.8) |
| Kristeller, 2014 (40) | Adults with overweight & obesity who binge eat & are concerned about their weight (66% meet criteria for Binge eating disorder) | Local advertisements (unspecified type) requesting individuals who binge eat & concerned about their weight | Inclusion criteria not explicitly mentioned; all had some symptoms of binge eating disorder; *Exclusion criteria*: purging behaviour/ bulimia nervosa | Caucasian: 129 (86%); African American: 20 (13%); Hispanic: 1 (1%) | Two-thirds met DSM IV criteria for Binge Eating Disorder (BED). Others met some but not all BED criteria. | NR |
| Lee, 2017 (36) | Adults (25-65 years) with BMI 28-45 kg/m^2^ | Advertisements (unspecified type) | BMI 28-45 kg/m^2^; aged 25-65 years | White: 47 (89%); Black: 6 (11%) | NR | High school and some college: 37%; Bachelor’s degree and some college: 19%;  Completed grad school: 44% |
| McKee, 2014 (42) | Adults with overweight & obesity who have a weight loss goal & no health conditions requiring medical supervision of diet or exercise. | Email bulletin sent to university hospital & non-academic university staff, students & community members | BMI >25 who have a weight loss goal & no health conditions requiring medical supervision of diet or physical activity | White: 30 (54%); South Asian: 13 (24%); Black: 7 (13%); Chinese: 3 (6%); Other: 2 (3%) | NR | University honours degree: 43% |
| Miller, 2012 (69; 89) | Adults with BMI>27 kg/m^2^ & type 2 diabetes | Local medical practices, the university newswire, radio & Internet advertisements, & community flyers | BMI ≥27 kg/m^2^; aged 35-65 years, type 2 diabetes mellitus ≥1 year, HbA_1c_ ≥7%, not requiring insulin therapy | White: 40 (77%); Black: 11 (21%); Asian: 1 (2%) | Type 2 diabetes | Bachelor’s degree or higher: *Intervention*: 48%;  *Control*: 60% |
| Palmeira, 2017 (37; 70) | Adult women (18-55 yrs) with overweight & obesity(BMI 25 kg/m^2^) without binge eating, enrolled in nutritional treatment  for weight loss in primary care units & Hospitals from Coimbra's  district, Portugal | Recruited at medical care units on day of appointment by a clinical psychologist (member of the research team) | BMI ≥25 kg/m^2^; aged 18-55 years; Female | NR | NR | Mean years of education: 15.6 (SD=3.2) |
| Raja-Khan, 2017 (32; 61) | Women with overweight or obesity (with & without polycystic ovary syndrome) | Medicine & obstetrics & gynaecology clinics at Penn State Hershey Medical Center, paper, radio & website advertisements | BMI ≥25 kg/m^2^; aged ≥18 years; Female.  If on medication must be on stable regimen for ≥6 weeks prior | White: 77 (89%); Black: 5 (6%); Other: 4 (5%) | Prediabetes: 35(40.7%)  Type 2 diabetes: 21 (24.4%)  PCOS: 32 (37.2%) | NR |
| Smith, 2017 (45) | Post-menopausal women (50-70 yrs) with obesity | Fliers & email announcements | BMI ≥30 kg/m^2^; aged 50–70 years; postmenopausal; ability to participate in study for 1 year; fluency in English; ability to walk ≥10 min without stopping | NR | NR | Not clear (mean of the categories of years of education was mentioned, which is difficult to interpret). |
| Spadaro, 2017 (46; 90) | Aged >18 with BMI 25-40 kg/m^2^ | Local newspaper, newsletters, television & radio advertisements | BMI 25-40 kg/m^2^; aged ≥18 years | Caucasian: 36 (78%); African American: 10 (22%) | NR | Bachelor’s degree or higher: 46%; some college: 33%; HS/ Vocational: 22% |
| Studies with an intervention using ACT | |  |  |  |  |  |
| Butryn, 2017 (54; 91) | Adults (18-70 yrs) with BMI 27-45 kg/m^2^ | Community through radio, newspaper, local websites & postcards. Outlets expected to reach a racially diverse audience targeted | BMI 27-45 kg/m^2^; aged 18-70 years; ability to engage in physical activity; completion of a 7-day food diary; 2 pre-randomization screening/assessment visits | White: 186 (66%); Black/ African American: 83 (29%); Other: 14 (5%) | NR | Graduate or professional degree:    **BT**: 37 (42%);  **BT+E**: 46 (50%);  **BT+EA**: 35 (35%) |
| Fletcher, 2011(30) | Current or past students in a weight loss/maintenance programme | Recruited from weight loss programme (meal replacements & 12 weeks of classes) at University of Nevada Medical School (Health Management Resources (HMR)) or flyer mailed to previous students | Aged ≥18 years; current or past enrolment in HMR; interest in increasing physical activity; fluent in English | Caucasian: 64 (89%); Other: 8 (11%) | NR | NR |
| Forman, 2013 (71) | Adults (21-65 years) with BMI 27-40 kg/m^2^ & have the ability to engage in PA | Local newspaper & radio advertisements, & recruitment flyers mailed to community health-care providers | BMI 27-40 kg/m^2^; aged 21-65 years; ability to engage in physical activity | Caucasian: 80 (62%); African American: 31 (25%); Asian: 2 (2%); Hispanic: 5 (4%) | Depression | NR |
| Forman, 2016 (72; 92) | Adults (18-70 yrs) with BMI 27-50 kg/m^2^ | Referrals from local primary care physicians & newspaper & radio advertisements | BMI 27-50 kg/m^2^; aged 18-70 years | Caucasian: 134 (71%); African American: 47 (25%); Asian: 2 (1%); Hispanic: 7 (4%) | NR | NR |
| Lillis, 2016 (62; 73; 93) | Adults with obesity & high levels of internal disinhibition | Newspaper advertisements & direct mailings. | BMI 30-50 kg/m^2^; aged 18-70 years; a score of ≥5 (women) or ≥4 (men) on internal disinhibition subscale of the Eating Inventory | Black/African American: 8 (5%); Hispanic: 10 (6%); Asian: 2 (1%); Caucasian (non-Hispanic): 142 (88%) | NR | High school/ G.E.D. 7.5%; Some college/ vocational: 28%; Bachelor’s degree: 36.5%;  Graduate or professional: 28% |
| Sairanen, 2017 (44; 60; 63; 94) | Adults with overweight & obesity reporting stress | Advertisements in local newspapers | BMI 27–34.9 kg/m^2^; perceived psychological stress (≥3/12 points in General Health Questionnaire; access to computer with internet connection | Caucasian: 219 (100%) | NR | Upper secondary education: 51·6%;  University degree: 45·7% |
| Studies with an intervention using CFT | |  |  |  |  |  |
| Loader, 2013 (41) | Adult outpatients accessing treatment at a specialist NHS weight management clinic between July & December 2012 | Attendees of weight management clinic between June & December 2012. Approached by researchers. | BMI ≥30 kg/m^2^; aged 18-65 years; fluent in English | White: 25 (75%); Black: 4 (12%); Indian: 3 (8%); Other: 1 (3%) | NR | NR |
| Studies with an intervention using DBT | |  |  |  |  |  |
| Adler, 2008 (66) | Adults (≥18) with BMI>27 kg/m^2^ & binge eating disorder | NR | BMI ≥27 kg/m^2^; aged ≥18 years; meeting criteria for binge eating disorder or sub-threshold binge eating disorder; available & committed to attend treatment & follow up; fluency in English | Caucasian: 11 (65%); African American: 1 (6%); Asian/ Pacific Islander: 2 (12%); Latino: 3 (18%) | Depression: 65% | High school grad: 5.9%; Some college: 41.2%; College: 17.0%; Graduate school: 35.3% |

DBT=dialectical behavioural therapy; ACT=acceptance & commitment therapy; MBCT=mindfulness-based cognitive behavioural treatment; CFT=compassion-focussed therapy; NR=Not reported; PCOS: Polycystic ovary syndrome.

**Table S2b, Participant characteristics of included pre-post studies**

| 1st author (year) | Study population | Recruitment method | Inclusion criteria | Race/Ethnicity; N (%) | Co-morbidities | Education |
| --- | --- | --- | --- | --- | --- | --- |
| Studies with an intervention using MBCT | | | | | | |
| Braun, 2012 (28) | Adults with overweight and obesity who attended an integrated weight loss programme | Enrolees from 6 successive cohorts of Integrated Weight Loss (IWL) program from July 2008 to May 2009 by email | BMI ≥25 kg/m^2^; attended IWL program | Caucasian: 37 (100%) | NR | NR, just mentioned "most had high levels of education & income" |
| Braun, 2016 (65) | **Study one:** overweight, yoga-experienced women  **Study two:** overweight, yoga-naïve women | Advertisements in a North-eastern rural region of the United States | BMI ≥25 kg/m^2^; aged ≥18 years; Female; fitness to walk 2 miles at a moderate pace.  **Study one**: practiced yoga ≥once per for 6 months  **Study two**: participated in ≤4 yoga classes in lifetime | **Study one**: Caucasian: 22 (100%)  **Study two**: Caucasian: 20 (95%) | NR | **Study one**: 84.2% had 4-year college degree or professional training beyond college.  **Study two**: 4-year college degree or professional training beyond college (71.4%) |
| Chung, 2016 (38) | African American Adults with BMI ≥25kg/m2 who have completed active treatment for Breast Cancer (stages I-III).  Mean BMI: 35.13 (3.97)  Female: 100  Mean age: 50.14 (9.0) | Oncology practices, community churches, & health fairs | BMI ≥25kg/m^2^; Self-described Black or African American racial background; history of breast cancer (stages I–III); completed active treatment ≥3 months prior (with exception of aromatase inhibitors/tamoxifen); ability to speak English; medical clearance from oncologist or primary care provider | African American: 29 (100%) | Breast cancer stage I-III (completed active treatment) | Education at or above a Bachelor’s degree: 64%;  Some college education:18% |
| Dalen, 2010 (29) | Adults with BMI ≥30 kg/m^2^ | Recruited through local YMCA | BMI ≥30 kg/m^2^ | Caucasian: 6 (60%); Hispanic: 2 (20%); Native American: 2 (20%) | NR | 1 participant completed high school; 9 had some college. |
| Hamel, 2010 (64) | Adults with BMI 25-35 kg/m^2^ | Waiting list for a previously offered programme & local newspaper advertisements | Inclusion criteria not explicitly mentioned. | NR | NR | Secondary education:60% |
| Hanson, 2019 (77) | Adults (≥18 years) with BMI ≥35 kg/m2; | Patients newly referred to the tier 3 hospital weight-management service who were amenable to attending group sessions | BMI ≥35 kg/m2; aged ≥18 years; proficient English language skills. |  |  |  |
| Lundgren, 2003 (78) | Adults (≥18 years) with BMI ≥25 kg/m^2^ | Advertisement in local news magazine, referral from behavioural weight loss program, flyers & brochures posted in YMCAs & yoga studio | BMI ≥25 kg/m^2^; aged ≥18 years; no diagnosis of type 2 diabetes or thyroid disorder | NR | NR | NR |
| Studies with an intervention using ACT | |  |  |  |  |  |
| Andalcio, 2018 (76) | Adults (≥18 years) with BMI ≥30 kg/m^2^ | During routine visit to medical practice | Adults (≥18 years) with BMI ≥30 kg/m2; English speaking; males & females | African American: 23 (100%) | NR | U.S. Census data (2010) reported socioeconomic status of residents in this area was mainly middle class |
| Boucher, 2016 (34) | Premenopausal women (40-50 years) with BMI>26.5 & low intuitive eating scores | Recruitment materials circulated through local health promotion & social service networks, low socioeconomic neighbourhoods, & organizations servicing Pacific & Māori populations, & promoted in newspaper article | BMI≥26.5 kg/m^2^; aged 40-50 years; Female; premenopausal; regularly accessed internet; low Intuitive Eating Scale; communicate in English; able to engage in PA (walk at leisurely pace for 10 minutes or more) | New Zealand European: 32 (81.1%); Maori: 4 (10.8%); Pacific: 1 (2.7%); Other: 2 (5.4%) | NR | University: 56.8%; Technical/trade/polytechnic: 27%; Secondary or less: 16.2% |
| Bradley, 2017 (79; 95) | Adults (18-70 years) who had weight-loss surgery within 1.5 years & had regained weight lost | Patients from the University of Pennsylvania bariatric surgery program. Advertisements through community flyers & craigslist | Aged 18-70 years; weight-loss surgery in at least 1.5 years prior to enrolment; more than 10% weight regain of maximum weight loss or 5% of their minimum weight post-surgery & weight regain lasting for at least 3 months prior to enrolment | White: 16 (80%); African American: 4 (20%) | NR | NR |
| Forman, 2009 (80) | Employees of Urban University & associated medical centre with a BMI >25kg/m^2^ | Employees at an urban university & its associated medical center using flyers, email, university newsletter, & website advertisements | BMI ≥25 kg/m^2^; agreement not to join another weight loss program for the duration of the study | Caucasian: 15 (52%); African American: 14 (48%) | NR | High School Education: 100%; College Graduates: 54% |
| Niemeier, 2012 (75) | Adults (21 -70 years) with overweight or obesity (BMI 27- 40 kg/m2) scoring high on internal disinhibition | Newspaper advertisements that sought individuals interested in weight loss who also have trouble controlling their eating when stressed | BMI 27-40 kg/m^2^; aged 21-70 years; a score of ≥5 on the internal disinhibition subscale of the Eating Inventory | Non-Hispanic White: 19 (90%); Hispanic: 1 (4.8%); Other: 1 (4.8%) | NR | NR |
| Studies with an intervention using DBT | |  |  |  |  |  |
| Gallé, 2017 (39) | Caucasian women (>18 years) with borderline personality disorder who had had laparoscopic adjustable gastric banding or laparoscopic gastric bypass | Candidates for laparoscopic adjustable gastric banding or laparoscopic gastric bypass interviewed before surgery for presence of Borderline Personality Disorder in hospital? | Aged ≥18 years; candidates for laparoscopic adjustable gastric banding or laparoscopic gastric bypass; met criteria for borderline personality disorder | Caucasian: 153 (100%) | Borderline personality disorder | NR |
| Roosen, 2012 (43) | Adults with obesity who are emotional eaters | Referred directly from their GP to the eating disorders outpatient clinic of the mental health care centre | BMI 30-40 kg/m^2^; aged 18-65 years; high levels of emotional eating (Dutch Eating Behaviour Questionnaire score ≥2.38) | NR | NR | NR |

**Table S3a; Intervention characteristics of included randomised controlled trials**

| 1^st^ Author (year) | | Country | | | Setting | | Treatment content | | | Mode of delivery | | Group or individual | Intervention duration | | No. of times intervention delivered | | Intensity of intervention delivery | | Duration of each session | Materials | | Intervention providers | |
| --- | --- | --- | --- | --- | --- | --- | --- | --- | --- | --- | --- | --- | --- | --- | --- | --- | --- | --- | --- | --- | --- | --- | --- |
| Studies with an intervention using MBCT | | | | | | | |  |  | |  | | |  | |  | |  | | |  | |  |
| Blevins, 2008 (33) | | | USA | | | NR | Group: Mindfulness meditation techniques & guided exercises addressing weight, shape & eating-related self-regulatory processes; body awareness scan; introductory mindful yoga; instructed to follow low-calorie & low-fat diet (1200-1500 kCals/day & 25% total fat); recommended 30 mins MVPA PA ≥5 days/week; taught goal-setting, self-monitoring, stimulus control, problem solving, social support; reinforcement strategies & relapse prevention skills.  Manual: Session plans with learning objectives, strategies & self-monitoring materials.  Home: Meditation practice between sessions using CD; “Mini-meditations” a few moments during day | | | Face to face (with home-based tasks) | | Group | 8 weeks | | 8 | | Weekly session | | 2 hrs (30 mins check-in, 30 mins didactic training, 15 mins group activity & discussion & 45 mins mindfulness training) | 2 manuals & meditation CD | | Advanced graduate students in clinical & health psychology with experience in behavioural weight loss treatment.  Group leaders had 6-week training course & weekend retreat with instructor. Met for weekly planning sessions. | |
| Carpenter, 2017 (74) | | | USA | | | Home | Telephone: Calls 1 & 2: Goal setting, tracking food & weight & nutrition advice. Call 2 onwards: Introduced treatment rationale & basic mindfulness concepts. Calls 1-11: Started with 60-second mindfulness exercise; coach check-in re: weight, food tracking & PA. Topics of calls: mindfulness meditation, mindfulness of everyday activities, mindful eating, acceptance of thoughts & emotions, & self-compassion.  Email: Brief educational eLessons | | | Telephone & email | | Individual | 6 months | | 11 telephone counselling sessions & unlimited inbound support calls; 10 e-Lessons | | 11 telephone calls during 6 months at participant's convenience (weekly or biweekly) | | Calls 1-4: av. 30 mins; calls 5-11: av. 20 mins | Printed program guide; Fit-bit Zip activity tracker; tape measure; food journal; Fitbit aria wireless scale; emailed links to eLessons (links to resources & exercises) | | 2 calls with RD & other calls by health coaches (or RD) with >200 hours of training.  Mindful coaches received additional training | |
| Daubenmier, 2011 (67; 87) | | | USA | | | NR | Group: Mindfulness practice & discussion (body scan, yoga, sitting meditation, loving kindness, or forgiveness), review of progress & guided meditations (mindful eating practices, identification of emotional & eating triggers, self-acceptance & inner wisdom).  Supplementary meditations: Awareness of negative emotions, & loving kindness & forgiveness towards others. 2-hr nutrition & PA session aimed at moderate weight loss midway through intervention.  Retreat day: Practice meditations & potluck meal to practice mindful eating skills.  Home: Up to 30 mins/day of mindfulness practices 6 days/week & before & during meals. | | | Face to face (with home-based tasks) | | Group | 4 months | | Nine 2.5-hr classes; one 7-hr silent day retreat of guided meditation practice; one 2-hr nutrition & exercise information session | | Weekly session | | Nine 2.5-hour sessions; one 7-hr silent day of guided  meditation practice (retreat day); one 2-hr nutrition & exercise information session | NR | | NR | |
| Daubenmier, 2016 (68; 88) | | | USA | | | University | Group: Mindful walking: in class instruction, book, DVD & workbook on principles from Chi Walking ; Mindfulness training for stress management (e.g. sitting meditation, loving kindness & yoga postures), discussion, & review of progress & challenges over previous week; Mindful eating practices: guided mediations, teaching practices of enhance awareness & self-regulation of physical hunger, stomach fullness, taste satisfaction, food cravings, emotions, & other eating triggers; Dietary: recommended healthy food choices emphasizing modest calorie reduction (500 kcal/day). PA: increasing daily & moderate-intensity PA.  Home: Meditation practice up to 30 min daily/6 days a week, eating meals mindfully & mini meditations. | | | Face to face (with home-based tasks) | | Group | 5.5 months. | | 16 sessions, 1 all-day session | | 12 weekly sessions, 3 biweekly & 1 monthly & 1 all-day session over 5.5 months | | 16 sessions of 2-2.5 hrs & 1 all-day session (6.5 hrs) | Binder, food records, calorie counting book, Calorie King, pedometers, Chi Walking book, DVD & workbook, CDs with meditation instructions for home practice | | Led by 1 of 3 mindfulness meditation instructors & co-led by same RD  (except for 1 cohort). | |
| Davis, 2008 (35) | | | USA | | | University of Pittsburgh Physical Activity & Weight Management Research Center | Group: 30 min didactic behavioural lessons & group discussions based on social cognitive theory; topics on strategies for adopting & maintaining positive diet & PA behaviours; weekly recipes; supplemental printed materials (worksheets, recipes & home activities); weigh-in; review of diet diaries; reduction of fat & calorie intake; increase to 300 mins PA a week; 30 mins supervised PA; PA diary; mindfulness mediation focusing on improving eating & PA behaviours. Lesson topics: breathing exercises, visualization, focused eating & walking meditations, progressive relaxation, yoga, & personal awareness. | | | Face to face (with home-based tasks) | | Group | 24 weeks | | 24 | | Weekly | | SBWL meeting: 30 mins. Supervised PA: 30 mins.  Mindfulness: additional 15-30 mins | Recipes, book, printed materials (e.g. worksheets & home activities, food diary, exercise diary)  Handouts & CD/DVD for non-attenders. | | Nutritionist, exercise physiologist or health educator with experience in weight loss interventions, graduate student with expertise in mindfulness mediation | |
| Goldbacher, 2016 (31) | | | USA | | | NR | Group: Weeks 1-2: Review model of emotional eating & process & rationale for tracking emotional eating episodes; weeks 3–19: additional skills to increase emotional awareness & regulation & improve ability to tolerate/decrease negative experiences & make intentional choices; Week 20: review & discuss all skills; review of task from previous week; introduce new skill; setting calorie & PA goals; info on behaviour weight loss strategies  Home: Practice emotional-eating specific skills with CD recording; monitor food & PA; written summaries of topics; instructions for setting goals between sessions; written ‘‘skill builders’’ containing tasks for upcoming week | | | Face to face (with home-based tasks) | | Group | 20 weeks | | 20 | | Weekly | | 90 mins | CD of emotional eating skills; written summaries of sessions; instructions for setting & achieving goals | | Masters & doctoral-level clinicians. | |
| Kristeller, 2014 (40) | | | USA | | | Indiana State or Duke University | Group: Topics related to normalising eating patterns & overcoming binge eating; teaching & practice of general (e.g. breath/open awareness) mindfulness meditation, guided eating meditations & “mini-meditations”; group discussion; potluck meal; body awareness; self-acceptance practices; healing self-touch  Home: Practice mediations; mindful eating exercises; tasks relating to week’s theme | | | Face to face (with home-based tasks) | | Group | 21 weeks | | 12 | | 9 weekly sessions & 3 monthly boosters | | 1.5 hr (sessions 1 & 6 were 2hr) | Session manuals; audio recordings for guided meditations. | | 2 facilitators: ≥1 licensed mental health provider at doctoral/ masters level & clinical or counselling psychology doctoral student/ masters level professional with experience | |
| Lee, 2017 (36) | | | USA | | | University of Kentucky | Group: Weight loss programme based on obesity & diabetes guidelines; supplement based on mindfulness book  Home: Weekly tasks | | | Face to face (with home-based tasks) | | Group | 12 weeks | | 12 | | Weekly | | 60 mins | Mindfulness book | | RD | |
| McKee, 2014 (42) | | | UK | | | University seminar room & emails | Group: Development of key self-regulatory skills (delay gratification, thought control, goal setting skills, self-monitoring, mindfulness & coping skills); group discussions Home: Interactive tasks; emails with practice tasks | | | Face to face (with home-based tasks) | | Group workshops & email | 8 weeks | | 2 workshops & 8 emails | | Week 1 & 3: workshops. Weekly emails | | Week 1: 3 hrs; Week 3: 1.5 hrs | 8 emails with tasks | | Lead researcher | |
| Miller, 2012 (69; 89) | | | USA | | | NR | Group: Guided meditations: on experiences & emotions associated with food intake (main focus), cultivating awareness of the distinction between physical & emotional hunger cues, social pressures to eat & preferences regarding food choices; medical nutrition therapy info; encouragement to engage in PA & mindful movement (no specific diet or PA goals).  Home: Practice meditations with a CD-ROM 6 days/week & mini-meditations before meals | | | Face to face (with home-based tasks) | | Group | 6 months | | 10 (& 2 follow-up sessions at month 4 & 6) | | 8 weekly & 2 biweekly | | 2.5 hrs (follow up sessions: 90 mins) | Written intervention protocol & 2 CD-ROMs for home use | | Dietitian & social worker with training in mindful meditation | |
| Palmeira, 2017 (37; 70) | | | Portugal | | | Primary care units & hospitals | Group: Psychoeducation on eating, weight & emotions to decrease shame & self-criticism; values & committed actions towards healthier life; acceptance of unwanted internal experiences, cognitive diffusion & distress tolerance skills; mindfulness promoted in all sessions; self-compassion to tackle weight stigma & self-criticism patterns (including loving kindness meditation & CFT exercises); mindfulness exercises  Home: Weekly practices of mindfulness & compassion exercises using audio-files | | | Face to face (with home-based tasks) | | Group | 3.5 months | | 12 | | 10 weekly & 2 biweekly | | 30 min  of shared experience & 5-min mindfulness practice | ACT books & manuals for eating & weight issues; participant manual & exercise sheets; audio files | | Clinical psychologist with training in contextual-behavioural therapies & clinical psychology master student | |
| Raja-Khan, 2017 (32; 61) | | | USA | | | Unclear | Group: Stress reduction; body scan; breath mediation; yoga; mindful walking; mindful listening; mindful interpersonal communications; unpleasant events calendar; group discussions; resources (written guidelines on diet & PA)  Home: 25-30 mins of daily practice | | | Face to face (with home-based tasks) | | Group | 8 weeks | | 9 | | 8 weekly & 1 retreat | | 2.5 hrs (& 1 6-hr retreat) | Printed guidelines on diet & PA | | Instructor with professional MBSR training & 9 years’ experience training others | |
| Smith, 2017 (45) | | | USA | | | University | Group: Group discussion; mindfulness meditation & group eating exercises; examined hunger & satiety cues, qualities of foods craved, emotional & cognitive states associated with eating; eating exercise with foods; basic yoga & walking meditations (2 classes) & increased PA recommended;  Refresher sessions: Meditation, eating exercise & group discussion.  Home: 9-min centering meditation CD (focus on breath meditation) & eat 1 mindful meal daily. | | | Face to face (with home-based tasks) | | Group | 6 weeks active intervention; 10 monthly refreshers | | 16 | | 6 weekly sessions, 10 monthly follow ups | | 6 weekly sessions: 2 hrs; monthly refreshers: 1 hr. | Written materials; recorded meditation exercises; meditation CD | | Medical doctor & trained mindfulness stress reduction instructor | |
| Spadaro, 2017 (46; 90) | | | USA | | | University | Group: Self-monitoring, goal setting, problem solving, mastery skills to influence self-efficacy, social support, relapse prevention strategies; taught mindfulness-based stress reduction strategies (e.g. body scan, walking meditation, yoga, 3-min check-in & loving kindness); hunger, taste, & satiety sitting meditations with focused awareness & tips for emotional & stress eating & food choices; mindfulness of body & awareness of negative thoughts on PA; diet (reduce calories/day) & PA (moderate intensity exercise 5 days/week) advice  Home: Practice & record mindfulness daily; PA & diet diaries | | | Face to face (with home-based tasks) | | Group | 6 months | | Weekly for 6m | | Weekly | | 30 mins SBWP, 30 mins supervised exercise & 30 mins mindfulness | Sample meal plans, menus, & recipes; The Calorie King® Calorie, Fat,  & Carbohydrate Counter book; 5 mindfulness meditation & 3 yoga CDs & written materials for home practice | | Author trained & experienced in mindfulness; Master’s/PhD level graduate students in exercise physiology, nutrition (or related); trained & supervised by senior staff | |
| Studies with an intervention using ACT | | | | | | | | | | | | | | | | | | | | | | | |
|  | | | | | | | |  |  | |  | | |  | |  | |  | | |  | |  |
| Butryn, 2017 (54; 91) | | USA | | | Community  research clinic on university campus | | Group: Self-monitoring, goal setting, problem solving, relapse prevention, stimulus control; calorie goals based on weight, PA goal of 250 mins/week; change home food environment; willingness to engage in weight control behaviours when experiencing uncomfortable internal experiences; discussions & exercises on clarifying what is important in life; enhance psychological flexibility to choose value-driven behaviour; emphasis on ACT skills to support environmental change & behavioural skills & target  Home: Calorie & PA goals; modify home environment | | | Face to face (with home-based tasks) | | Group | 12 months | | 26 sessions | | Months 1-4: weekly; Months 5-6: biweekly; months 7-12: monthly | | 75 mins | Manual | | Masters/doctoral degree in psychology with experience conducting behaviour weight loss interventions | |
| Fletcher, 2011 (30) | | USA | | | University Center for Nutrition & Metabolism | | Group: Topics on: framing the problem, values (mediation), barriers, reason-giving, control, acceptance, distress tolerance (discussion & breath holding exercise), defusion & mindfulness exercises, self-stigma; invited to share a commitment with group from workshop. | | | Face to face | | Group | 1 day | | 1 | | 1 day | | 6-hr workshop (30 mins for lunch & 2 5-10 min breaks) | NR | | Doctoral level graduate students with >2 years of experience in ACT & had weekly supervision with ACT developer | |
| Forman, 2013 (71) | | USA | | | Unclear | | Group: Nutritional education; stimulus control, behaviour shaping, behaviour analysis & relapse prevention strategies; identification of triggers for overeating & barriers to PA & problem solving; obtain social support for behavioural changes; ACT strategies focusing on non-compliance: erosion of commitment, distress intolerance & mindless eating; identification of weight-related goals emanating from personal life values; strategies integrated into hand-outs, tip-sheets & problem-solving techniques; increase awareness of moment-by-moment behaviour choices & reflecting one’s ultimate goals; ‘urge surfing’; demonstrations & practice of diffusion & uncoupling; metaphors & experiential exercises on becoming more present-centred & aware  Home: Daily self-monitoring of calories & brisk walking | | | Face to face (with home-based tasks) | | Group | 40 weeks | | 30 | | Weeks 1-20: weekly; Weeks 21-40: bi-weekly | | 75-mins | Pedometer | | Doctoral students & experienced clinical psychologists | |
| Forman, 2016 (72; 92) | | USA | | | NR | | Group: Individual check-ins; skills presentation; skill building exercises; nutrition & PA education; stimulus control (e.g., removal of problematic foods); behaviour analysis (e.g., reviewing factors leading to lapse); relapse prevention (e.g., identifying triggers for overeating/ sedentary behaviour); problem solving (e.g. identifying barriers to healthy eating & activity & developing solutions to overcome); Social support (e.g. communicating needs, building positive support); values clarification; ongoing commitment; mindful decision-making training; psychological acceptance & willingness to experience less pleasurable or comfortable states.  Home: Self-monitoring & calorie & PA goals | | | Face to face (with home-based tasks) | | Group | 12 months | | 25 | | Weekly for 16 sessions; biweekly for 5 sessions; monthly for 2 sessions; & bi-monthly for 2 sessions | | 75 mins | Course manual | | Doctoral level clinician with average of 4.8 years’ experience delivering weight loss treatment. Trainees acted as group co-leaders | |
| Lillis, 2016 (62; 73; 93) | | USA | | | Weight Control & Diabetes Research Centre | | Group: Stimulus control, problem solving, & goal setting; Acceptance & mindfulness strategies; Mindful awareness of/detachment from problematic thoughts; Acceptance of unwanted emotions & food cravings; Values clarification techniques; Commitment to values-consistent behaviour in the presence of difficult thoughts, feelings, & cravings  Home: Work towards calorie goal; Gradual increase of PA; Self-monitoring of weight & food intake | | | Face to face (with home-based tasks) | | Group | 1 year | | 32 sessions | | Months 1-6: Weekly; Months 6-9: bi-weekly; Months 9-12: Monthly | | 1 hr | Fat/calorie guidebook, food diaries | | PhD exercise physiologists,  & master’s level nutritionists, leaders with training & experience running weight loss groups, received 2-day training & weekly supervision | |
| Sairanen, 2017 (44; 60; 63; 94) | | Finland | | | NR | | Group: Topics: my life here & now; values & mindful living; value-based actions & barriers; the observing self & acceptance; mindful eating; summary & reflection; experiential exercises (e.g. mindfulness & acceptance exercises & individual activation through value work); pair & group discussions.  Home: Tasks related to topic of the session. | | | Face to face  Mobile | | Group  Individual | 8 weeks | | 6 | | 6 sessions | | 90 mins | Coach manual; participant workbook with session summaries exercises & individual notes | | Psychologist trained in ACT approach | |
|  | |  | | |  | |  | | |  | |  |  | |  | |  | |  |  | |  | |
| Studies with interventions using CFT | | | | | | | |  |  | |  | | |  | |  | |  | | |  | |  |
| Loader, 2013 (41) | UK | | | Weight management clinic outpatients | | | Group: Psycho-education; information on factors affecting weight (e.g. diet & PA); guided self-help bibliotherapy focussed on a compassionate mind approach to overeating.  Home: 1 chapter every two weeks with 1 support telephone call relevant to that chapter every 2 weeks | | | Face to face & telephone | | Initial group session & individual support calls. | 6 months | | 1 group session & 12 support calls | | 1 chapter every 2 weeks with 1 support telephone call every 2 weeks | | Initial group session: 90 mins, further sessions not specified; support call: 15 mins | Self-help manual | | Trainee Clinical Psychologists trained in CFT | |
| Studies with interventions using DBT | | | | | | | |  |  | |  | | |  | |  | |  | | |  | |  |
| Adler, 2008 (66) | USA | | | Conference room in University | | | Group: Mindfulness exercises, “chain analysis” of behaviours with hierarchical focus on treatment interfering behaviours (e.g. binge eating, eating off-plan & urges); orlistat (60 mg, 90 tablets) to be taken 3 times a day & vitamin supplement  Home: Print or online access to the self-help behavioural weight-loss programme. Topics: explanation of Alli program; an eating plan with target goals; enlisting support network, beginning the eating plan, adherence tips & using the capsules; info regarding PA & tools for making plan; info on dealing with cravings, sabotage, plateaus & remaining motivated; eating guide; daily journal; forum. | | | Face to face & online/ print self-help | | DBT in group sessions | 12 weeks | | 12 DBT sessions | | Weekly DBT sessions | | DBT sessions=2 hours | Self-help manual, orlistat/alli | | NR | |

DBT=dialectical behavioural therapy; ACT=acceptance & commitment therapy; MBCT=mindfulness-based cognitive behavioural treatment; CFT=compassion-focussed therapy; NR=Not reported

**Table S3b; Intervention characteristics of included pre-post design studies**

| 1^st^ Author (year) | Country | Setting | Treatment content | Mode of delivery | Group or individual | Intervention duration | No. of times intervention delivered | Intensity of intervention delivery | Duration of each session | Materials | Intervention providers | |
| --- | --- | --- | --- | --- | --- | --- | --- | --- | --- | --- | --- | --- |
| Studies with interventions using MBCT | | | | | | | | | | | | |
| Braun, 2012 (28) | USA | Residential retreat (Kripalu Center for Yoga & Health) | Group: Set context for acceptance, self-compassion, mindfulness, self-care; fitness walk (mindful fitness); holistic health & weight loss lecture; mindful/intuitive eating workshop; integrative weight loss yoga class; self-care (e.g. walking, journal, sauna); get fit: tone & strengthen mindful exercise; nutrition & natural weight management lecture; Kripalu yoga; menu planning workshop; body Image: developing self-compassion workshop; whole foods cooking demo; obstacles: commitment & strategies workshop; 90 mins Kripalu yoga daily. | Face to face (with free time for self-care) | Group | 5 days | 5 day residential | 5 day residential. Sunday: 7.30-9pm. Monday, Tuesday & Thursday: 7am-5.45pm. Wednesday: 7am-9pm. Friday: 6.30am-10.30am | Excluding breaks: 6.5 hrs daily. Includes 90 mins yoga daily. | NR | NR | |
| Braun, 2016 (65) | USA | Yoga training & retreat centre | Group: Workshop sessions: 30 mins of gentle yoga & 90 mins of lectures & activities relating to Ayurveda-inspired & yogic theory & practices; additional yoga sessions: 90-min Kripalu Yoga class (breathing exercises, postures, final relaxation & meditation) & 30-min sharing circle  Home:  *Study 1:* Engage in one of the following home practices for 30 mins/week: (1) brisk outdoor walk; (2) deep breathing; (3) meditation; or (4) yoga postures.  *Study 2:* 2 30-min sessions of gentle yoga & either 15-min sessions: (1) brisk outdoor walk; (2) breathing exercises; or (3) meditation. | Face to face (with home-based tasks) | Group | 10 weeks | *Study 1*: 14 sessions  *Study 2*: 20 sessions | *Study 1*: 10week, 14-session curriculum  (weeks 1 -4 twice-weekly, 2-hr workshop & yoga sessions; weeks 5-10 yoga sessions  *Study 2*: revised 10-week, 20- session curriculum | Workshops: 30 mins yoga, 90 mins lectures & activities  Yoga sessions: 90mins yoga class & 30min sharing circle | Audio recordings & paper handouts | 2 trained  Ayurvedic lifestyle practitioners, senior Kripalu Yoga dean of an Ayurvedic  training facility | |
| Chung, 2016 (38) | USA | Community & oncology practice | Individual: Dietary counselling (calorie, carbohydrate, protein, fat reduction)  Group: Mindful eating; reinforcement of healthy eating principles (e.g. planning a balanced eating plan & identifying hidden fats); body scan; mindful meditation; applying mindfulness to difficult eating & social situations; mindful cooking session; mindful walking; how to apply mindfulness to maintenance.  Telephone: Support calls. | Face to face & telephone | Individual (diet counselling) & group (mindfulness) | 24 weeks (12 week active intervention; 12 week follow up) | 18 (6 bi-weekly sessions mindfulness, 6-biweekly sessions diet counselling, 6 sessions telephone) | Weekly for first 12 weeks (bi-weekly sessions alternate), bi-weekly for weeks 13-24 | 120 mins | Workbook | Diet counselling delivered by RD. Telephone support by study staff. Person delivering mindfulness NR. | |
| Dalen, 2010 (29) | USA | YMCA | Group: Sitting meditation, eating meditations, light yoga, walking meditation, and group discussion. Topics on hunger & satiety cues & each class has eating exercise in different context (hungry, full, alone, social)  Home: listen to a recorded 10-min mindfulness meditation daily & engage in mindful eating; encouraged to increase their general PA by 5-10%/week. | Face to face (with home-based tasks) | Group | 6 weeks | 6 sessions | Weekly | 2 hrs | Written materials & CDs for home practice | Intervention developer (author) | |
| Hamel 2010 (64) | NL | NR | Group: Sessions 1-5: Info on mindfulness; techniques to slow down eating rate; practiced & discussed mindfulness exercises (e.g. body scan); hunger & satiety cues; practiced exercises. Sessions 6-10: meal consumed collectively to practice with ‘JUM’ plate  Home: Exercises; practice with JUM plate | Face to face (with home-based tasks) | Group | NR | 10 sessions | Weekly or alternating weeks | NR | ‘JUM’ interactive plate (measures amount of food taken & tells eater how long to chew) | Instructors | |
| Hanson 2019 (77) | UK | Specialist weight-management service at hospital | Group: Identification of methods for weight loss & maintenance; group discussion of past attempts; education & discussion on complexity of weight loss (e.g. environmental); introduction to mindfulness & mindful eating with exercise & discussion; introduction to compassionate mind therapy & application to eating related behaviour; share experiences of stigma & critical patterns of thought; development of mindful & compassionate planning & management of relapse; motivational video to support discussions on long-term weight maintenance | Face to face (with home-based tasks) | Group | 8 weeks | 4 | Alternating weeks | 90 mins per session | Powerpoints, flipcharts, motivational video | Specialist dietitians, doctors & psychologist | |
| Lundgren, 2005 (78) | USA | NR | Group: Weeks 1-6: mindfulness-based stress reduction programme & DBT (e.g. dealing with barriers, mindfulness of breath, yoga, non-judgement, preparing for future).  Weeks 7-20: traditional behavioural treatment (e.g. goal setting, self-monitoring, diet & PA info) enhanced with mindfulness exercises (e.g. observing, describing & willingness skills)  Home: practice meditation and yoga exercises | Face to face (with home-based tasks) | Group (unclear) | 20 weeks | 20 | Weekly | 1.5-2 hrs per session | Mindfulness meditation practice tapes & supplies for in-session demonstrations (e.g. food) | Yoga instructor, nutritionist, exercise specialist | |
| Studies with an intervention using ACT | | | | | | | | | | | | |
| Andalcio 2018 (76) | USA | Primary care family practice | Sessions: Wk 1: Obesity education on associated diseases, lifestyle modifications (e.g. diet, PA & self-monitoring); set goals; instructions on using resources; printed information on healthy eating & lifestyle; received folders with goal & current weights/BMI & sample 1800 calorie diet plan; encouraged to engage in PA. Wk 8: Review of progress on diet, PA, stress & sleeping; additional info on diet modification; positive reinforcement & encouragement. Wk 16: Additional reinforcements & education; encouraged to continue lifestyle modifications; provided with resources  Telephone calls & text messages: Reviewed progress; education/counselling on diet (e.g. calorie counting & portion size) & PA; positive reinforcement; encouragement; answered questions | Face to face & telephone calls & texts | Individual (sessions unclear) | 16 wks | 16 | Weekly: 3 face to face sessions (wks 1, 8 & 16) & telephone calls or texts between sessions (13 total) | NR | Food & drink tracker, ‘Remember your Purpose Questionnaire’, BMI chart, printed program goals, sample 1800 calorie diet & lifestyle modification (diet & PA) chart, texts & telephone calls | Researcher (PhD candidate) | |
| Boucher, 2016 (34) | NZ | Online | Website modules (12): Ditch the Diet (self-permission to eat range of foods); Tuning into Hunger (recognizing physical hunger); Am I Full? (recognizing fullness); One Bite at a Time (mindfulness); Coping with cravings (acceptance); Emotional eating (emotional triggers to eat i.e. acceptance); Everybody deserves respect (focus on body functions; Dealing with pressures to diet (Handling pressures to diet or engage in fat talk); Taming the inner critic (cognitive defusion); Get Active Your Way; Fine tuning food choices (Selecting healthier food options without feeling deprived); Staying on track. | Internet (website delivery & home tasks) | Individual | 14 weeks | 12 modules | 12 modules completed over 14 weeks. New module available once previous completed. | 15-20 mins to complete | Website, email or text reminders, paper prototype | Website developed by researchers, pretesting with target audience & created by web-developer | |
| Bradley, 2017 (79; 95) | USA | Online | Website modules: Emphasis on willingness to experience less pleasurable foods & aversive internal experiences; taught strategies to increase willingness; mindful decision-making; clarification & commitment to living life in accordance with one’s values; behavioural techniques for weight loss (i.e., self-monitoring, stimulus control, portion control, psychoeducation); interactive exercises/challenges (e.g. quizzes); discussion board (topics posted by staff); examples of others utilizing ABT skills  Telephone: Clarify content of session; discuss utilization of skills; review homework; feedback regarding weight loss & food records  Home: Weekly assignments; record food intake daily using MyFitnessPal; record weights & average daily calories in an online spreadsheet - self-populated a graph | Internet (website delivery) & telephone (with home-based tasks) | Individual | 10 weeks | 10 | Weekly (biweekly phone support) | Average time interacting with module: 26 mins. 20 min phone call every 2 weeks | E-learning software suite (Articulate) | Program coach' advanced graduate students with at least 1 year experience delivering ABTs for weight control | |
| Forman, 2009 (80) | USA | Place of employment (university & affiliated medical centre) | Group: Diet & PA info; self-monitoring of calorie intake; environmental influences; monitoring weight; meal planning; problem solving strategies; stimulus control; body image; long-term weight goals; acceptance; willingness; values clarification; workability & flexibility; limitations of experiential control; mindful eating & PA; urge surfing; defusion vs fusion; committed action; metaphors; practice of exercises  Home: Daily food monitoring & exercises based on lessons | Face to face (with home-based tasks) | Group | 12 weeks | 12 sessions | Weekly | 1 hr | Session manual created by the investigators, food monitoring forms | PhD level psychologist & doctoral student in clinical psychology | |
| Niemeier, 2012 (75) | USA | NR | Group: prescriptions for weight loss (e.g. reduction of calories & from fat; increase PA); self-monitoring; stimulus control; problem solving; assertiveness training; goal setting; relapse prevention; strategies to help cope with internal experiences (identification of how their eating & activity behaviours fit with their values; acceptance techniques to notice & tolerate unpleasant thoughts & feelings; cognitive defusion skills to; committed action strategies focusing on values during heightened distress & discomfort) | Face to face (not reported whether home-based tasks were involved) | Group | 6 months | 24 | Weekly | 1 hr | NR | PhD-level clinical psychologist, clinical psychology intern & master's-level nurse with expertise in behavioural weight loss | |
| Studies with an intervention using DBT | | | | | | | | | | | | |
| Gallé, 2017 (39) | Italy | NR | Group: Topics: increasing behavioural capabilities; improving motivation for skilful behaviour; assuring generalization of gains to the natural environment; structuring the treatment environment to reinforce functional behaviours; enhancing therapist capabilities and motivation to treat patients effectively  Individual (unclear): Evaluation of interpersonal relationships to reinforce individual functioning. Weeks 1-16: aim for therapeutic alliance, control impulsive & self-destructive conduct & promote an initial improvement of symptoms. Weeks 17 onwards: strengthen initial results & develop stable & appropriate interpersonal relationships.  Home: Tasks & telephone consultation offered | Face to face (with home-based tasks & telephone, if required) | Group & individual (telephone & unclear if other individual component) | 12 months | 52 | Weekly | 60 mins | NR | | Advanced therapist , psychologist |
| Roosen, 2012 (43) | NL | NR | Individual: Initial pre-treatment interview to orient to treatment goals (reducing eating pathology by teaching emotion regulation skills), emphasise importance of maintaining body weight, developing & maintaining a healthy eating pattern & obtaining sufficient PA). Group: Teach adaptive emotion regulation skills through mindfulness, emotion regulation & distress tolerance; 2 final sessions review & relapse prevention. | Face to face | Individual pre-treatment interview & group | 20 weeks | 20 | Weekly | 2 hrs | Therapist manual for treating binge translated into Dutch; brochure of the National Nutrition Centre | | 2 trained co-therapists |

DBT=dialectical behavioural therapy; ACT=acceptance & commitment therapy; MBCT=mindfulness-based cognitive behavioural treatment; CFT=compassion-focussed therapy; PA=physical activity; NR=Not reported; MVPA=moderate vigorous physical activity; RD=registered dietician

**Table S4a; Risk of bias judgements for randomised controlled trials (RoB 2.0)**

| 1^st^ author (year) | Bias arising from the randomization process | Bias due to deviations from intended intervention | Bias due to missing outcome data | Bias in measurement of the outcome | Bias in selection of the reported result | Overall |
| --- | --- | --- | --- | --- | --- | --- |
| Adler, 2008 (66) | Some concerns | Some concerns | Some concerns | Low | Some concerns | Some concerns |
| Blevins, 2008 (33) | Some concerns | Low | Some concerns | Low | Some concerns | Some concerns |
| Butryn, 2017 (54; 91) | Some concerns | Low | Low | Low | Low | Low |
| Carpenter, 2017 (74) | Low | Low | Low | High | Some concern | High |
| Daubenmier, 2011 (67; 87) | Low | Low | Low | Low | Some concern | Low |
| Daubenmier, 2016 (68; 88) | Low | Low | Low | Low | Low | Low |
| Davis, 2008 (35) | Some concerns | Some concerns | Low | Low | Some concern | Some concerns |
| Fletcher, 2011 (30) | Some concerns | Some concerns | Low | Low | Some concerns | Some concerns |
| Forman, 2013 (71) | Some concerns | Low | Some concerns | Low | Low | Some concerns |
| Forman, 2016 (72; 92) | Some concerns | Low | Low | Low | Low | Low |
| Goldbacher, 2016 (31) | Some concerns | Some concerns | Low | Low | Some concerns | Some concerns |
| Kristeller, 2014 (40) | Some concerns | Low | High | Low | Some concerns | Some concerns |
| Lee, 2017 (36) | High | Some concerns | High | Low | Some concerns | High |
| Lillis, 2016 (62; 73; 93) | Some concerns | Low | Low | Low | Low | Low |
| Loader, 2013 (41) | Low | Low | Low | High | Some concerns | High |
| McKee, 2014 (42) | High | Some concerns | Some concerns | Some concerns | Some concerns | High |
| Miller, 2012 (69; 89) | Some concerns | Low | Some concerns | Low | Some concerns | Some concerns |
| Palmeira, 2017 (37; 70) | Some concerns | Some concerns | Low | Low | Some concerns | Some concerns |
| Raja-Khan, 2017 (32; 61) | Low | Low | Some concerns | Low | Low | Low |
| Sairanen, 2017 (44; 60; 63; 94) | Low | Low | Low | Low | Low | Low |
| Smith, 2017 (45) | Some concerns | Some concerns | Low | Low | Some concerns | Some concerns |
| Spadaro, 2017(46; 90) | Some concerns | Some concerns | Low | Low | Some concerns | Some concerns |

**Table S4b; Risk of bias judgements for non-randomised studies (ROBINS-I)**

| 1st author (year) | Bias due to confounding | Bias in selection of participants into the study | Bias in classification of interventions | Bias due to deviations from intended interventions | Bias due to missing data | Bias in measurement of outcomes | Bias in selection of the reported result | Overall |
| --- | --- | --- | --- | --- | --- | --- | --- | --- |
| Andalcio, 2018 (76) | N/A | Low | Low | Low | Low | Moderate | Moderate | Moderate |
| Boucher, 2016 (34) | N/A | Low | Low | Low | Moderate | Low | Moderate | Moderate |
| Bradley, 2017 (79; 95) | N/A | Moderate | Low | Moderate | Serious | Serious | Moderate | Serious |
| Braun, 2012 (28) | N/A | Moderate | Low | Low | Serious | serious | Moderate | Serious |
| Braun, 2016 (65) | N/A | Moderate | Low | Low | Moderate | Serious | Moderate | Serious |
| Braun, 2016 (65) | N/A | Moderate | Low | Low | Moderate | Serious | Moderate | Serious |
| Chung, 2016 (38) | N/A | Moderate | Low | Low | Low | Serious | Moderate | Serious |
| Dalen, 2010 (29) | N/A | Moderate | Low | Low | Low | Moderate | Moderate | Moderate |
| Forman, 2009 (80) | N/A | Moderate | Low | Low | Serious | Low | Moderate | Serious |
| Galle, 2017 (39) | Serious | Low | Low | Serious | Low | Moderate | Moderate | Serious |
| Hamel, 2010 (64) | N/A | Moderate | Low | Low | Serious | Serious | Moderate | Serious |
| Hanson, 2019 (77) | N/A | Low | Low | Low | Low | Low | Moderate | Moderate |
| Lundgren, 2003 (78) | N/A | Moderate | Low | Low | Moderate | Serious | Moderate | Serious |
| Niemeier, 2012 (75) | N/A | Moderate | Low | Low | Low | Low | Moderate | Moderate |
| Roosen, 2012 (43) | N/A | Moderate | Low | Low | Low | Low | Moderate | Moderate |

**Table S5a; GRADE assessment at different follow-up time point (DIRECT EVIDENCE)**

| Comparison | Risk of bias | Inconsistency | Indirectness | Imprecision | Publication bias | Total | Quality of evidence |
| --- | --- | --- | --- | --- | --- | --- | --- |
| Post-intervention |  |  |  |  |  |  |  |
| Third-wave CBT vs. No/min | 0 | 0 | 0 | 0 | 0 | 0 | High |
| Third-wave CBT vs. SBT | -1 | 0 | 0 | 0 | 0 | -1 | Moderate |
| MBCT vs. No/min | 0 | 0 | 0 | 0 | 0 | 0 | High |
| ACT vs. No/min | 0 | 0 | 0 | 0 | 0 | 0 | High |
| SBT vs. No/min | -1 | 0 | 0 | 0 | 0 | -1 | Moderate |
| MBCT vs. SBT | -1 | 0 | 0 | 0 | 0 | -1 | Moderate |
| ACT vs. SBT | 0 | 0 | 0 | 0 | 0 | 0 | High |
| CFT vs. SBT | -2 | 0 | -1 | -1 | 0 | -4 | Very low |
| 3-months |  |  |  |  |  |  |  |
| Third-wave CBT vs. No/min | 0 | 0 | 0 | -1 | 0 | -1 | Moderate |
| Third-wave CBT vs. SBT | -1 | 0 | 0 | 0 | 0 | -1 | Moderate |
| MBCT vs. No/min | 0 | 0 | 0 | -1 | 0 | -1 | Moderate |
| MBCT vs. SBT | -1 | 0 | 0 | 0 | 0 | -1 | Moderate |
| ACT vs. SBT | -1 | 0 | 0 | -1 | 0 | -2 | Low |
| 6-months |  |  |  |  |  |  |  |
| Third-wave CBT vs. No/min | -1 | 0 | 0 | 0 | 0 | -1 | Moderate |
| Third-wave CBT vs. SBT | -1 | 0 | 0 | -1 | 0 | -2 | Low |
| MBCT vs. No/min | -1 | 0 | 0 | 0 | 0 | -1 | Moderate |
| SBT vs. No/min | -1 | 0 | 0 | 0 | 0 | -1 | Moderate |
| MBCT vs. SBT | -1 | 0 | 0 | 0 | 0 | -1 | Moderate |
| ACT vs. SBT | 0 | 0 | 0 | 0 | 0 | 0 | High |
| CFT vs. SBT | -2 | 0 | -1 | -1 | 0 | -4 | Very low |
| 9-months |  |  |  |  |  |  |  |
| Third-wave CBT vs. No/min | 0 | 0 | 0 | 0 | 0 | 0 | High |
| Third-wave CBT vs. SBT | -1 | 0 | 0 | 0 | 0 | -1 | Moderate |
| MBCT vs. No/min | -1 | 0 | 0 | 0 | 0 | -1 | Moderate |
| ACT vs. No/min | 0 | 0 | 0 | 0 | 0 | 0 | High |
| SBT vs. No/min | -1 | 0 | 0 | 0 | 0 | -1 | Moderate |
| MBCT vs. SBT | -1 | 0 | 0 | 0 | 0 | -1 | Moderate |
| ACT vs. SBT | -1 | 0 | 0 | 0 | 0 | -1 | Moderate |
| 12-months |  |  |  |  |  |  |  |
| Third-wave CBT vs. SBT | 0 | 0 | 0 | 0 | 0 | 0 | High |
| MBCT vs. SBT | 0 | 0 | 0 | 0 | 0 | 0 | High |
| ACT vs. SBT | 0 | -1 | 0 | 0 | 0 | -1 | Moderate |
| 18-months |  |  |  |  |  |  |  |
| Third-wave CBT vs. SBT | 0 | 0 | 0 | 0 | 0 | 0 | High |
| MBCT vs. SBT | 0 | 0 | 0 | 0 | 0 | 0 | High |
| ACT vs. SBT | 0 | 0 | 0 | 0 | 0 | 0 | High |
| 24-months |  |  |  |  |  |  |  |
| Third-wave CBFT vs. SBT | 0 | 0 | 0 | 0 | 0 | 0 | High |
| ACT vs. SBT | 0 | 0 | 0 | 0 | 0 | 0 | High |
| 36-months |  |  |  |  |  |  |  |
| Third-wave CBT vs. SBT | 0 | 0 | 0 | 0 | 0 | 0 | High |
| ACT vs. SBT | 0 | 0 | 0 | 0 | 0 | 0 | High |

**Table S5b; GRADE assessment at different follow-up time point (INDIRECT EVIDENCE)**

| Comparison | First order loops | | Quality of evidence |
| --- | --- | --- | --- |
|  | **Pairwise comparisons** | **Quality** |  |
| Post-intervention |  |  |  |
| MBCT vs. No/min | MBCT vs. SBT | Moderate | Moderate |
|  | SBT vs. No/min | Moderate |  |
| ACT vs. No/min | ACT vs. SBT | High | Moderate |
|  | SBT vs. No/min | Moderate |  |
| CFT vs. No/min | SBT vs. No/min | Moderate | Very low |
|  | CFT vs. SBT | Very low |  |
| MBCT vs. SBT | SBT vs. No/min | Moderate | Moderate |
|  | MBCT vs. No/min | High |  |
| ACT vs. SBT | ACT vs. No/min | High | Moderate |
|  | SBT vs. No/min | Moderate |  |
| ACT vs. MBCT | ACT vs. SBT | High | Moderate |
|  | MBCT vs. SBT | Moderate |  |
| CFT vs. MBCT | MBCT vs. SBT | Moderate | Very low |
|  | CFT vs. SBT | Very low |  |
| CFT vs. ACT | CFT vs. SBT | Very low | Very low |
|  | ACT vs. SBT | High |  |
| 3-months |  |  |  |
| ACT vs. MBCT | MBCT vs. SBT | Moderate | Low |
|  | ACT vs. SBT | Low |  |
| 6-months |  |  |  |
| MBCT vs. No/min | SBT vs. No/min | Moderate | Moderate |
|  | MBCT vs. SBT | Moderate |  |
| ACT vs. No/min | ACT vs. SBT | High | Moderate |
|  | SBT vs. No/min | Moderate |  |
| CFT vs. No/min | CFT vs. SBT | Very low | Very low |
|  | SBT vs. No/min | Low |  |
| ACT vs. MBCT | ACT vs. SBT | High | Moderate |
|  | MBCT vs. SBT | Moderate |  |
| CFT vs. MBCT | CFT vs. SBT | Very low | Very low |
|  | MBCT vs. SBT | Moderate |  |
| CFT vs. ACT | ACT vs. SBT | High | Very low |
|  | CFT vs. SBT | Very low |  |
| 9-months |  |  |  |
| MBCT vs. No/min | SBT vs. No/min | Moderate | Moderate |
|  | MBCT vs. SBT | Moderate |  |
| ACT vs. No/min | ACT vs. SBT | Moderate | Moderate |
|  | SBT vs. No/min | Moderate |  |
| ACT vs. SBT | SBT vs. No/min | Moderate | Moderate |
|  | ACT vs. No/min | High |  |
| ACT vs. MBCT | MBCT vs. SBT | Moderate | Moderate |
|  | ACT vs. SBT | Moderate |  |
| 12-months |  |  |  |
| ACT vs. MBCT | MBCT vs. SBT | High | Moderate |
|  | ACT vs. SBT | Moderate |  |
| 18-months |  |  |  |
| ACT vs. MBCT | MBCT vs. SBT | High | High |
|  | ACT vs. SBT | High |  |

**Table S5c; GRADE assessment at different follow-up time point (OVERALL EVIDENCE)**

| **Comparison** | **Direct evidence quality** | **Indirect evidence quality** | **Mixed evidence quality** |
| --- | --- | --- | --- |
| **Post-intervention** |  |  |  |
| MBCT vs. No/min | High | Moderate | High |
| ACT vs. No/min | High | Moderate | High |
| CFT vs. No/min | - | Very low | Very low |
| MBCT vs. SBT | Moderate | Moderate | Moderate |
| ACT vs. SBT | High | Moderate | High |
| CFT vs. SBT | Very low | - | Very low |
| ACT vs. MBCT | - | Moderate | Moderate |
| CFT vs. MBCT | - | Very low | Very low |
| CFT vs. ACT | - | Very low | Very low |
| **3-months** |  |  |  |
| MBCT vs. No/min | Moderate | - | Moderate |
| ACT vs. No/min | - | - | - |
| CFT vs. No/min | - | - | - |
| MBCT vs. SBT | Moderate | - | Moderate |
| ACT vs. SBT | Low | - | Low |
| CFT vs. SBT | - | - | - |
| ACT vs. MBCT | - | Low | Low |
| CFT vs. MBCT | - | - | - |
| CFT vs. ACT | - | - | - |
| **6-months** |  |  |  |
| MBCT vs. No/min | Moderate | Moderate | Moderate |
| ACT vs. No/min | - | Moderate | Moderate |
| CFT vs. No/min | - | Very low | Very low |
| MBCT vs. SBT | Moderate | - | Moderate |
| ACT vs. SBT | High | - | High |
| CFT vs. SBT | Very low | - | Very low |
| ACT vs. MBCT | - | Moderate | Moderate |
| CFT vs. MBCT | - | Very low | Very low |
| CFT vs. ACT | - | Very low | Very low |
| **9-months** |  |  |  |
| MBCT vs. No/min | Moderate | Moderate | Moderate |
| ACT vs. No/min | High | Moderate | Moderate |
| CFT vs. No/min | - | - | - |
| MBCT vs. SBT | Moderate | - | Moderate |
| ACT vs. SBT | Moderate | Moderate | Moderate |
| CFT vs. SBT | - | - | - |
| ACT vs. MBCT | - | Moderate | Moderate |
| CFT vs. MBCT | - | - | - |
| CFT vs. ACT | - | - | - |
| **12-months** |  |  |  |
| MBCT vs. No/min | - | - | - |
| ACT vs. No/min | - | - | - |
| CFT vs. No/min | - | - | - |
| MBCT vs. SBT | High | - | High |
| ACT vs. SBT | Moderate | - | Moderate |
| CFT vs. SBT | - | - | - |
| ACT vs. MBCT | - | Moderate | Moderate |
| CFT vs. MBCT | - | - | - |
| CFT vs. ACT | - | - | - |
| **18-months** |  |  |  |
| MBCT vs. No/min | - | - | - |
| ACT vs. No/min | - | - | - |
| CFT vs. No/min | - | - | - |
| MBCT vs. SBT | High | - | High |
| ACT vs. SBT | High | - | High |
| CFT vs. SBT | - | - | - |
| ACT vs. MBCT | - | High | High |
| CFT vs. MBCT | - | - | - |
| CFT vs. ACT | - | - | - |

CBT=Cognitive behaviour therapy; MBCT=Mindfulness-based cognitive behaviour treatment; ACT=Acceptance & commitment therapy; CFT=Compassion-focussed therapy; No/min=No/minimal intervention; CI=Confidence interval.

**Table S6; Pooled effects estimates of third-wave cognitive behaviour therapies on weight change estimated from random-effects meta-analysis**

| Follow up^‡^ | Intervention | N | Effect* (95% CI) | I^2^ | τ^2^ |
| --- | --- | --- | --- | --- | --- |
| Post-intervention | 3rd-wave CBTs combined | 35 | -0.84 (-1.06*,* -0.62) | 93.4 | 0.41 |
|  | MBCT | 22 | -0.76 (-1.08*,* -0.45) | 93.4 | 0.53 |
|  | ACT | 11 | -1.04 (-1.35*,* -0.74) | 92.6 | 0.26 |
|  | DBT | 1 | -0.59 (-0.92*,* -0.26) | - | - |
|  | CFT | 1 | -0.19 (-0.65*,* 0.27) | - | - |
| 3-months | 3rd-wave CBTs combined | 17 | -0.77 (-1.10, -0.44) | 92.0 | 0.44 |
|  | MBCT | 13 | -0.78 (-1.18, -0.37) | 93.2 | 0.50 |
|  | ACT | 4 | -0.75 (-1.35, 0.15) | 87.5 | 0.33 |
| 6-months | 3rd-wave CBTs combined | 23 | -0.92 (-1.23, -0.62) | 94.7 | 0.51 |
|  | MBCT | 14 | -0.81 (-1.24, -0.38) | 94.8 | 0.63 |
|  | ACT | 7 | -1.31 (-1.63, -1.00) | 86.6 | 0.15 |
|  | DBT | 1 | -0.59 (-0.92, -0.26) | - | - |
|  | CFT | 1 | -0.19 (-0.65, 0.27) | - | - |
| 9-months | 3rd-wave CBTs combined | 6 | -0.97 (-1.47, -0.47) | 94.4 | 0.42 |
|  | MBCT | 3 | -1.01 (-2.39, 0.38) | 97.2 | 1.45 |
|  | ACT | 3 | -0.94 (-1.41, -0.48) | 91.3 | 0.20 |
| 12-months | 3rd-wave CBTs combined | 7 | -1.16 (-1.59, -0.73) | 94.6 | 0.30 |
|  | MBCT | 3 | -1.16 (-2.24, -0.07) | 95.7 | 0.88 |
|  | ACT | 3 | -1.36 (-1.76, -0.97) | 91.3 | 0.11 |
|  | DBT | 1 | -0.61 (-0.94, -0.28) | - | - |
| 18-months | 3rd-wave CBTs combined | 3 | -0.77 (-1.12, -0.43) | 84.2 | 0.08 |
|  | MBCT | 1 | -0.47 (-0.68, -0.25) | - | - |
|  | ACT | 2 | -0.93 (-1.24, -0.62) | 68.4 | 0.03 |
| 24-months | 3rd-wave CBTs combined | 2 | -0.69 (-0.98, -0.40) | 74.2 | 0.03 |
|  | ACT | 2 | -0.69 (-0.98, -0.40) | 74.2 | 0.03 |
| 36-months | 3rd-wave CBTs combined | 1 | -0.47 (-0.67, -0.27) | - | - |
|  | ACT | 1 | -0.47 (-0.67, -0.27) | - | - |

*Since these estimates are within-group changes (before and after the intervention), we used *standardised mean change* to avoid the confusion with the *standardised mean difference* conventionally reserved for between-group comparisons. ^‡^Time since baseline/randomisation unless otherwise specified; N=Number of intervention arms.

CBT=Cognitive behaviour therapy; MBCT=Mindfulness-based cognitive behaviour treatment; ACT=Acceptance & commitment therapy; CFT=Compassion-focussed therapy; DBT=Dialectical behavioural therapy; CI=Confidence interval.

**Table S7; Meta-regression analysis of the effects of third-wave cognitive behaviour therapies on weight management compared to standard behavioural treatment**

|  | *P-*value | | |
| --- | --- | --- | --- |
| Study characteristics | **Post-intervention** | **3 months*** | **6 months*** |
| Number of sessions | 0.323 | 0.873 | 0.222 |
| Number of sessions (<12 vs. ≥12) | 0.187 | 0.592 | 0.465 |
| Some face-to-face sessions | 0.651 | ‡ | 0.698 |
| Predominantly face-to-face sessions | 0.760 | 0.587 | 0.547 |
| Some group sessions | 0.651 | ‡ | 0.698 |
| Predominantly group sessions | 0.760 | 0.587 | 0.547 |
| Intervention length (<12 vs. ≥12 months) | 0.241 | 0.592 | 0.473 |
| Intervention length (<6 vs. ≥6 months) | 0.898 | 0.372 | 0.710 |
| Bias level (low, some concern, high) | 0.977 | 0.586 | 0.551 |

*Months since randomisation; ‡ Could not be examined due to collinearity.

**Table S8a; Attendance and adherence information of randomised controlled trials**

| 1st author (year) | Attendance | Adherence |
| --- | --- | --- |
| Studies with an intervention using MBCT | |  |
| Blevins, 2008 (33) | NR | Self-reported home-based meditation (MBCT): mean: 4 mins/wk (range: 0-45).  Mindful meals (MBCT): Mean: 5 meals/week (range: 0 to 8) |
| Carpenter, 2017 (74) | Mean call completion   - MBCT: 6.0 (SD=4.2); SBT: 6.6 (SD=3.7) | 6 month survey (MBCT) (90% response rate):   - 33.3% (n=15/45) practiced meditation multiple times/week - 26% (n=12/45) never/almost never practiced meditation |
| Daubenmier, 2011 (67; 87) | Class attendance:   - All participants: 68% | **MBCT:**   - 4 did not receive minimum treatment dose   Mean weekly mediation practice (based on ≥4 weeks of adherence logs):   - Participants who attended ≥1 class: 98 (79) mins - “As treated” participants: 108 (75) mins   Number of mindful meals/week:   - Participants who attended ≥1 class: 5.9 (4.4) - “As treated” participants: 6.5±4.2 mindful meals/week |
| Daubenmier, 2016 (68; 88) | NR | Home worksheet practice completion:   - MBCT: 12.2/16 (SD=4.5); SBT: 12.4/16 (SD=4.38)   MBCT (n; Mean (SD)):   - Meditation (total hrs): n=100; Mean (SD): 26.2 (18.7) - Meditation (days): n=100; Mean (SD): 68.6 (28.5) - Meditation (hrs/week): n=100; Mean (SD): 2.1 (1.2) - Mindful meals (total #): n=97; Mean SD: 158.1 (103.3) - Mindful meals (days): n=97; Mean (SD): 70.8 (30.9) - Mindful meals (#/week): n=97; Mean (SD): 12.0 (6.0) - Mini-meditations (total #): n=94; Mean (SD): 107.3 (80.4) - Mini-meditations (days): n=94; Mean (SD): 56.2 (29.7) |
| Davis, 2008 (35) | Attendance at weekly group sessions (% of total sessions over 6 months)   - Mean: 63.7±30.2% - Intervention: 70.1±27.7% (16.8±6.6 sessions) - SBT (Control a): 62.3±26.7% (13.7±5.9 sessions) - SBT + resistance training (Control b): 57.9±36.3% (12.7± 8.0 sessions)   Attendance at mindfulness mediation (n=23):   - Intervention: 62.3±31.4% (14.3±7.2)   Attendance at resistance training session (n=21)   - Control b: 49.1±35.7% (10.3±7.5 sessions) | Return of food diaries (% of total number of possible diaries over 6 months)   - Overall: 52.4±31.7% - Intervention: 58.5±28.5%; Control a: 51.6±29.9%; Control b: 47.1±36.7%   Home-based mindfulness meditation practice (self-report diary)   - Intervention: approx. 5.29±2.9 practices/week   Home-based four days/week resistance training practice (% of prescribed days) (self-report diary)   - Control b: 40.3±33.9% |
| Goldbacher, 2016 (31) | Mean number of attendances at groups:   - Intervention: 13.56±5.57; Control: 10.95±6.38 | NR |
| Kristeller, 2014 (40) | NR | - Mediation practice an average of 16.49 times over 6.15 days - Mean frequency increased over course of treatment. - At end of treatment: over 2h of sitting meditation per week and about one half hour of mini-meditations per week (across a total of 18.95 meditation times on 6.42 days). |
| Lee, 2017 (36) | NR | NR |
| McKee, 2014 (42) | Participant attendance at ‘self-regulation’ training: Baseline, n=30; Booster session (week 3) n=26;  Participant attendance at ‘Advice’ workshop: Baseline, n=30; Booster session: n=24 | - No significant differences between groups in rating adherence to the allocated program (p=0.89) or to their own dietary & PA practices (p=0.30) during intervention. |
| Miller, 2012 (69; 89) | Mean attendance at group sessions:   - Intervention: 7.0/10; Control: 6.5/10.0 | NR |
| Palmeira, 2017 (37; 70) | - 24/27 participants attended the majority of the 12 sessions (Mean=10.89±1.12) | NR |
| Raja-Khan, 2017 (32; 61) | NR | Average adherence (hours of classes taken/26 hours x 100) of participants who attended ≥1 session: Intervention: 73.1±28.5%; Control: 68.4±29.0% |
| Smith, 2017 (45) | Attendance at monthly refresher sessions (range)   - Intervention: 77-88%; Control: 61-100% | NR (states data not collected) |
| Spadaro, 2017 (46; 90) | Session attendance   - Intervention: 75.2% (18.0±5.9 sessions) - Control: 62.3% (13.7±5.9 sessions) | Food diaries returned   - Intervention: 62.9% (range 0–22); Control: 51.5% (range 1–24)   Average number of times mindfulness practiced per week (self-reported recall) (Intervention group)   - Number of times practiced per week: 5.29±2.9 days - 3 months: 23 min/week (n=21); 6 months: 35 min/week (n=18) |
| Studies with an intervention using ACT | |  |
| Butryn, 2017 (54; 91) | Number of sessions attended:   - Mean: 19.43/26 (SD=5.99) sessions (74.6%) - Across conditions, average number of sessions attended did not significantly differ.   Attendance rate: Mean (SD)   - ACT=0.76 (0.21); Control a (SBT)=0.74 (0.26); Control b (SBT+ Environment)=0.73 (0.23) | Fidelity to treatment condition (score out of 10):  Intervention=9.24  Control a=9.13  Control b=9.20 |
| Fletcher, 2011 (30) | One day workshop (n=36 randomised to intervention): 3 unable to attend due to scheduling conflicts, and 2 did not turn up |  |
| Forman, 2013 (71) | Mean number of sessions attended:   - ACT: 21.08/30 (5.47); SBT: 19.96/30 (8.02)   Attended ≥25 of the 30 groups (% of participants)   - ACT: 77.0%; SBT: 70.4% | NR |
| Forman, 2016 (72; 92) | Average number of sessions attended   - ACT=21.26/25 (5.85); SBT=20.88/25 (5.46)   Attended ≥18 sessions: ACT: 84.2%; SBT: 85.6% | NR |
| Lillis, 2016 (62; 73; 93) | Mean number of sessions attended   - ACT: 28.5/32; SBT: 28.7/32   Treatment completion (≥70% sessions attended) for both groups: 74% | Average weekly food and exercise diary completion: ACT: 60%; SBT: 61% |
| Sairanen, 2017 (44; 60; 63; 94) | Not applicable | - Median number of usage sessions 21 (IQR 12-35), usage days 15/58 (IQR 9.0-24) & usage weeks 7.0/8.0 (IQR 4.0-8.0). - Median total duration of use 4.7 (IQR 3.2-7.2) hrs & number of exercises performed 63 (IQR 46-98). - Median session duration 13.5 (IQR 9.8-17) mins. - Median completion percentage was 91% (IQR 64%-96%) |
| Studies with an intervention using CFT | |  |
| Loader, 2013 (41) | Attendance at sessions NR but intervention group received average of 8 telephone calls | NR |
| Studies with an intervention using DBT | |  |
| Adler, 2008 (66) | NR | NR |

DBT=dialectical behavioural therapy; ACT=acceptance and commitment therapy; MBCT=mindfulness-based cognitive behavioural treatment; CFT=compassion-focussed therapy #: Number; NR=Not reported; IQR= Interquartile range.

**Table S8b; Attendance and adherence information of pre-post studies**

| 1st author (year) | Attendance | Adherence |
| --- | --- | --- |
| Studies with an intervention using MBCT | | |
| Braun, 2012 (28) | NR (delivered as a five day retreat) | NR |
| Braun, 2016 (65) | **Study one:** Sessions attended (N=14): 9.6±3.15 (69%);  **Study two:** Sessions attended (N=20): 13.8±5.21 (69%) | NR for both studies |
| Chung, 2016 (38) | NR | NR |
| Dalen, 2010 (29) | N=1 participant missed one session | NR |
| Hamel 2010 (64)  Hanson 2019 (77) | NR  33/53 participants attended at least 3 of 4 sessions | NR  NR |
| Lundgren, 2005 (78) | NR | Mean number of meditation practices: first 6 wk: 7.4; last 14 wk: 4.2 |
| Studies with an intervention using ACT | |  |
| Andalcio 2018 (76)  Boucher, 2016 (34) | NR  NR | NR  Completion of modules   - All 12: 32%; 7 to 11: 30%; 1 to 6: 32%; none: 5%; - Median: 7.5 (IQR 2-12)   Median number of sessions: Median: 7 (Range: 1 to 66)  Median session duration: 12 mins & 54 seconds (range: 3m58s-100m8s)  Number of EAT entries over 14 weeks: Median: 7 (Range: 1-314) |
| Bradley, 2017 (79; 95) | - 12/20 participants completed 8 modules;   Time interacting with each module: 26.2±10.2 mins  Phone call completion: Program completers: 100%; Program utilizers: 77.5%  Time of each phone call (mean): 16.5±3.6 mins  Use of discussion board (optional): No participants | Complete food records 67.4% days of the program.  Treatment completers recorded food 74.8% of days |
| Forman, 2009 (80) | - 74% classes attended by completers | Number of sessions attended & food records/assignments submitted (60±24) |
| Niemeier, 2012 (75) | Average sessions attended: of 20.5/24 (±4.8) (86%) | NR |
| Studies with an intervention using DBT | |  |
| Gallé, 2017 (39) | Attendance rate >65% | NR |
| Roosen, 2012 (43) | NR | NR |

DBT=dialectical behavioural therapy; ACT=acceptance and commitment therapy; MBCT=mindfulness-based cognitive behavioural treatment; CFT=compassion-focussed therapy #: Number; NR=Not reported; IQR= Interquartile range.

**Figure S1; Ranking plot showing the probability of each of the evaluated interventions of ranking the best to the worst intervention**

**Figure legends:** No/min=No/minimal intervention; SBT=Standard behavioural treatment; MBCT=Mindfulness-based cognitive behaviour treatment; ACT=Acceptance and commitment therapy; CFT=Compassion-focussed therapy.

**Figure S2; Pooled effects estimates of third-wave cognitive behaviour therapies on secondary outcomes estimated from random-effects meta-analysis at the earliest measurement post- intervention**

**Figure legends:** *For these variables, a desired change would be to the right of the dotted line while it would be to the left of the dotted line for other variables; N=Number of intervention arms.

CBT=Cognitive behaviour therapy; HbA_1c_=Glycated haemoglobin; LDL=Low-density lipoprotein; HDL=High-density lipoprotein; CI=Confidence interval.

**Figure S3; Effects on secondary outcomes comparing third-wave cognitive behaviour therapies and no/minimal intervention from random-effects pairwise meta-analysis at the earliest measurement post-intervention**

**Figure legends:** *Estimates to the right of the dotted line indicate a desired change in favour of third-wave CBTs, while it is to the left of the dotted line for other variables.

Estimates reported from one study are for completeness and may not be interpreted as the pooled intervention effects.

CBT=Cognitive behaviour therapy; MBCT=Mindfulness-based cognitive behaviour treatment; ACT=Acceptance and commitment therapy; CFT=Compassion-focussed therapy; SMD=Standardised mean difference; CI=Confidence interval.

AAQW=Acceptance and Action Questionnaire for Weight; BDI-II=Beck Depression Inventory- II (1996); DEBQ=Dutch Eating Behavior Questionnaire; FFMQ=Five Facets of Mindfulness Questionnaire; GBES=Gormally Binge Eating Scale; IES=Intuitive Eating Scale scores; PSS=Perceived Stress Scale; STAI-T=State-Trait Anxiety Inventory–Trait Scale; TFEQ=Three-Factor Eating Questionnaire; TFEQ–R18: Three-Factor Eating Questionnaire–R18.

**Brief description of the methods used:**

Where there are multiple studies reporting estimates of an effect of interest (e.g. intervention vs no intervention), **pairwise random effects meta-analysis** is a method to combine these effect estimates across studies. The method assumes that the true effect may vary between studies, and provides an estimate of the average effect and confidence interval, which takes into account both within-study precision and between-study variation. Statistical heterogeneity between studies is quantified using the I^2^ statistic, which is the percentage of the variability between effect estimates that is due to between-study variation rather than chance.

The method above requires each study to report an estimate for the comparison of interest (i.e. “direct evidence”). There may also be “indirect evidence” from other studies, e.g. estimates of the effect of the intervention of interest with a different comparator. **Network meta-analysis** is an extension of random effects meta-analysis which enables this indirect evidence to be incorporated, and also for several different interventions to be compared.

Where there is statistical heterogeneity between studies, **meta-regression** is a method that investigates the extent to which this heterogeneity can be explained by one or more study-level characteristics (e.g. duration of intervention). These characteristics can be included in the meta-regression model as either continuous or binary/categorical variables.

1. Linardon J, Fairburn CG, Fitzsimmons-Craft EE, Wilfley DE, Brennan L. The empirical status of the third-wave behaviour therapies for the treatment of eating disorders: A systematic review. *Clin Psychol Rev*. 2017;58:125-140. doi:10.1016/j.cpr.2017.10.005 [↑](#footnote-ref-1)
